# Supplementary material for: Oscillators that sync and swarm
Source: Nat Commun. 2017 Nov 15;8:1504. doi: 10.1038/s41467-017-01190-3 (PMC5686229; doi:10.1038/s41467-017-01190-3)
Supplement: Supplementary file 1 — Supplementary Information [file 41467_2017_1190_MOESM1_ESM.pdf]

SUPPLEMENTARY FIGURE 1

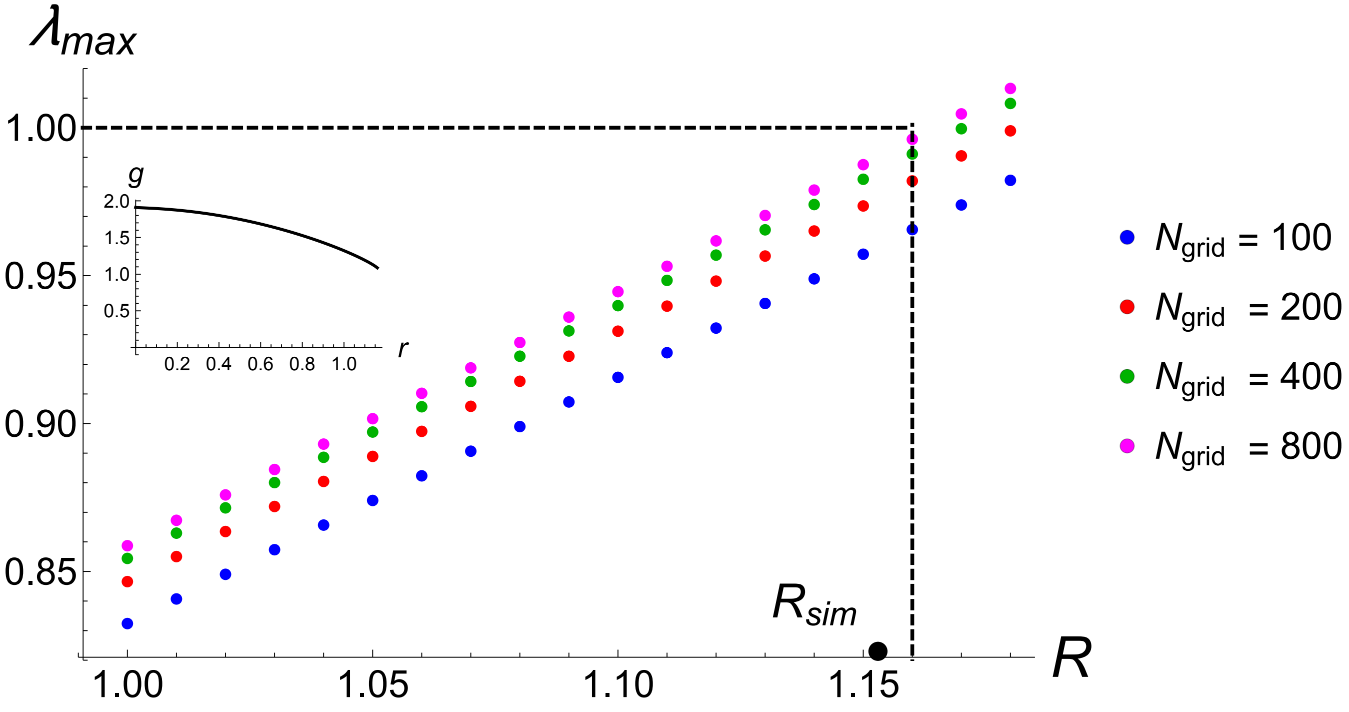

Supplementary Figure 1: **Determination of radius** R **of spatial density of static async state**. The largest eigenvalue of the matrix M_{ij} as defined by supplementary equation (9) is plotted versus R for different N_{grid}. The black, dashed lines show that the eigenvalue closest to 1 is achieved for a radius of $R = 1.16$. This is in reasonable with the radius $R_{sim} = 1.15$ measured from simulation data for $N = 1000$ swarmalators for $(J, K) = (0, -2)$ using python's solver 'odeint' for $T = 500$ time units and a stepsize of $dt = 0.1$. The inset shows the eigenvector g_i for $N_{grid} = 800$.

SUPPLEMENTARY FIGURE 2

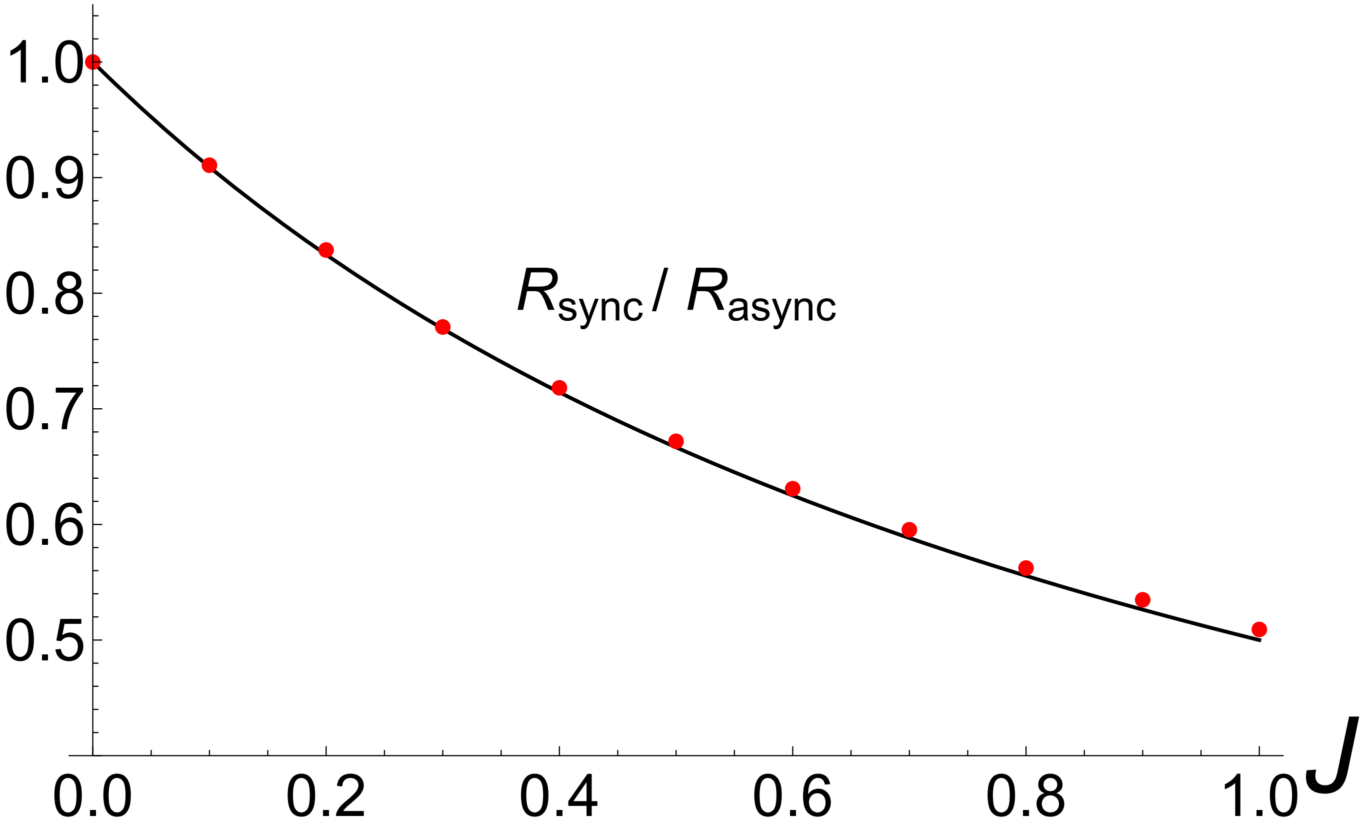

Supplementary Figure 2: **Radii of static sync and async states for unit vector attraction kernel**. Ratio of radius of static sync (for $K = 1$) and async states (for $K = -2$) versus J for $N = 800$ swarmalators for a unit vector attraction kernel $\mathbf{I}_{\rm att}(\mathbf{x}) = \mathbf{x}/|\mathbf{x}|$.

SUPPLEMENTARY FIGURE 3

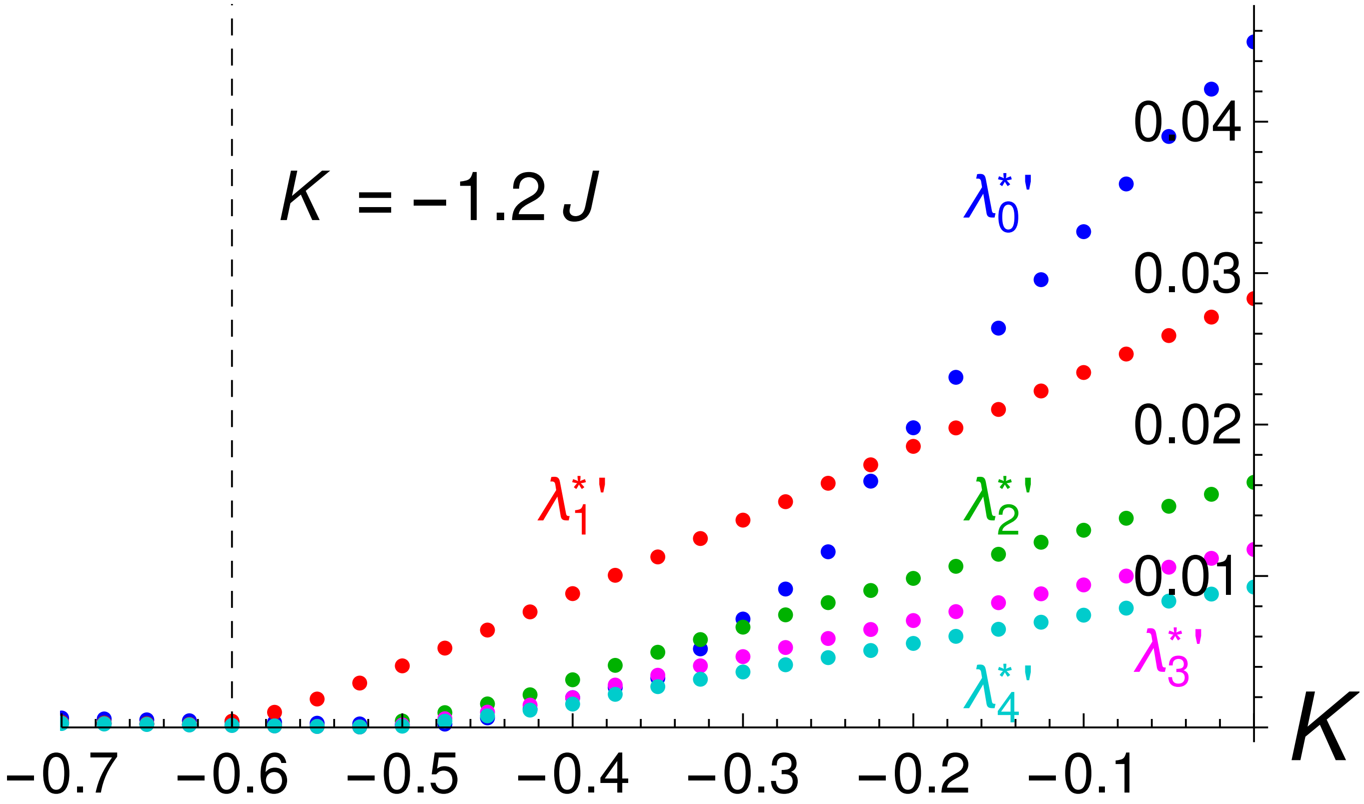

Supplementary Figure 3: **Spectrum**. The real part of the most unstable eigenvalue $\lambda_m^{*\prime}$ of the first five of the modes f_m calculated from supplementary equation (27) for $J = 0.5$. They are all small but positive for all K, indicating the static async state is unstable in the continuum limit $N \to \infty$. The dashed line marks the approximation to the critical coupling strength as per supplementary equation (29). The spectra for each mode were calculated by approximating the integral of the RHS of supplementary equation (27) using a Gaussian quadrature scheme with $N' = 200$ grid points and diagonalizing the resulting matrix. The upper limit of integration $R = 1.15$ was measured from simulations. The radial density $g(r)$ was determined numerically, as described in Supplementary Note 1. The kernels H_m in supplementary equation (27) for $m > 1$ were calculated numerically.

SUPPLEMENTARY FIGURE 4

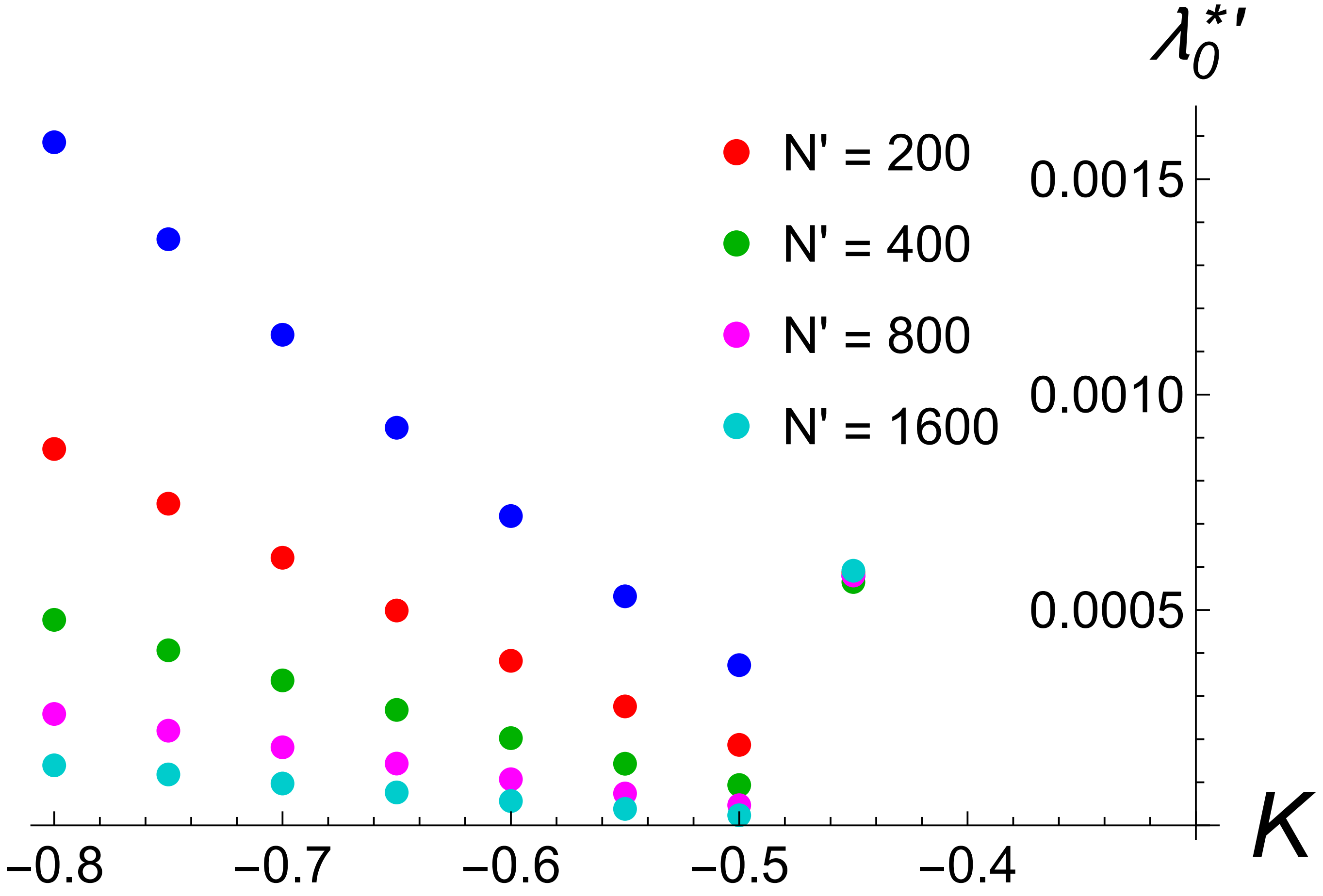

Supplementary Figure 4: **Finite size scaling of spectrum.** The real part of the most unstable eigenvalue of the first mode $\lambda_0^{*\prime}$ calculated from supplementary equation (27) for $J = 0.5$ for various N'. As can be seen, $\lambda_0^{*\prime}$ diminishes in magnitude for increasing N', but remains positive.

SUPPLEMENTARY FIGURE 5

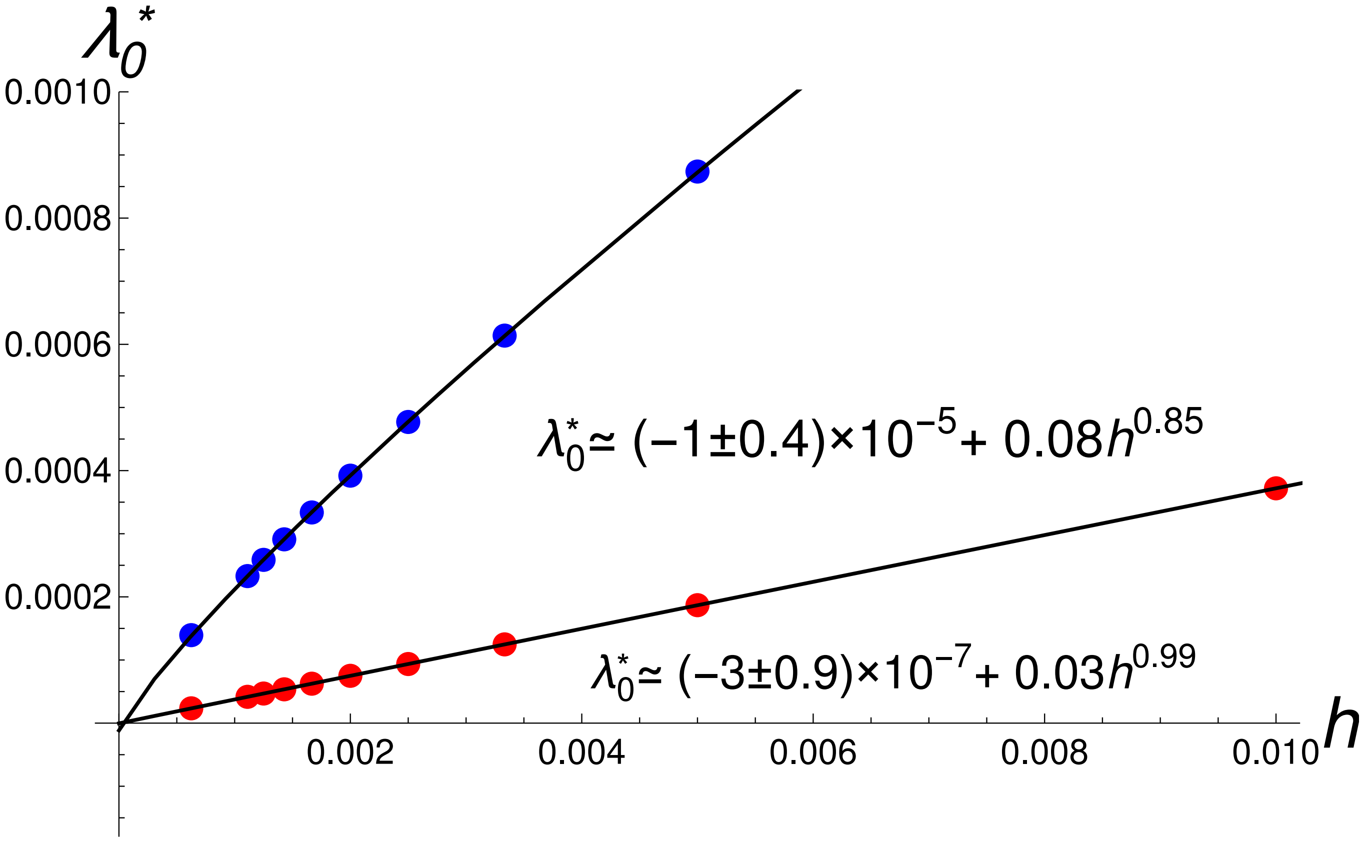

Supplementary Figure 5: **Finite size scaling of** $\lambda_0^{*\prime}$. Data are plotted versus $h = 1/N$ for $J = 0.5$. Red dots are for $K = -0.5$ and blue dots are for $K = -0.8$. To obtain the $h \to 0$ limit the data were fit to curves of the form $a + bh^c$ using Mathematica. The best fit parameters a, b, c are illustrated in the plot. As can be seen $\lambda_0^{*\prime}$ approaches small but negative values as $h \to 0$. Note also the different exponents of h for $K = -0.5, -0.8$.

SUPPLEMENTARY FIGURE 6

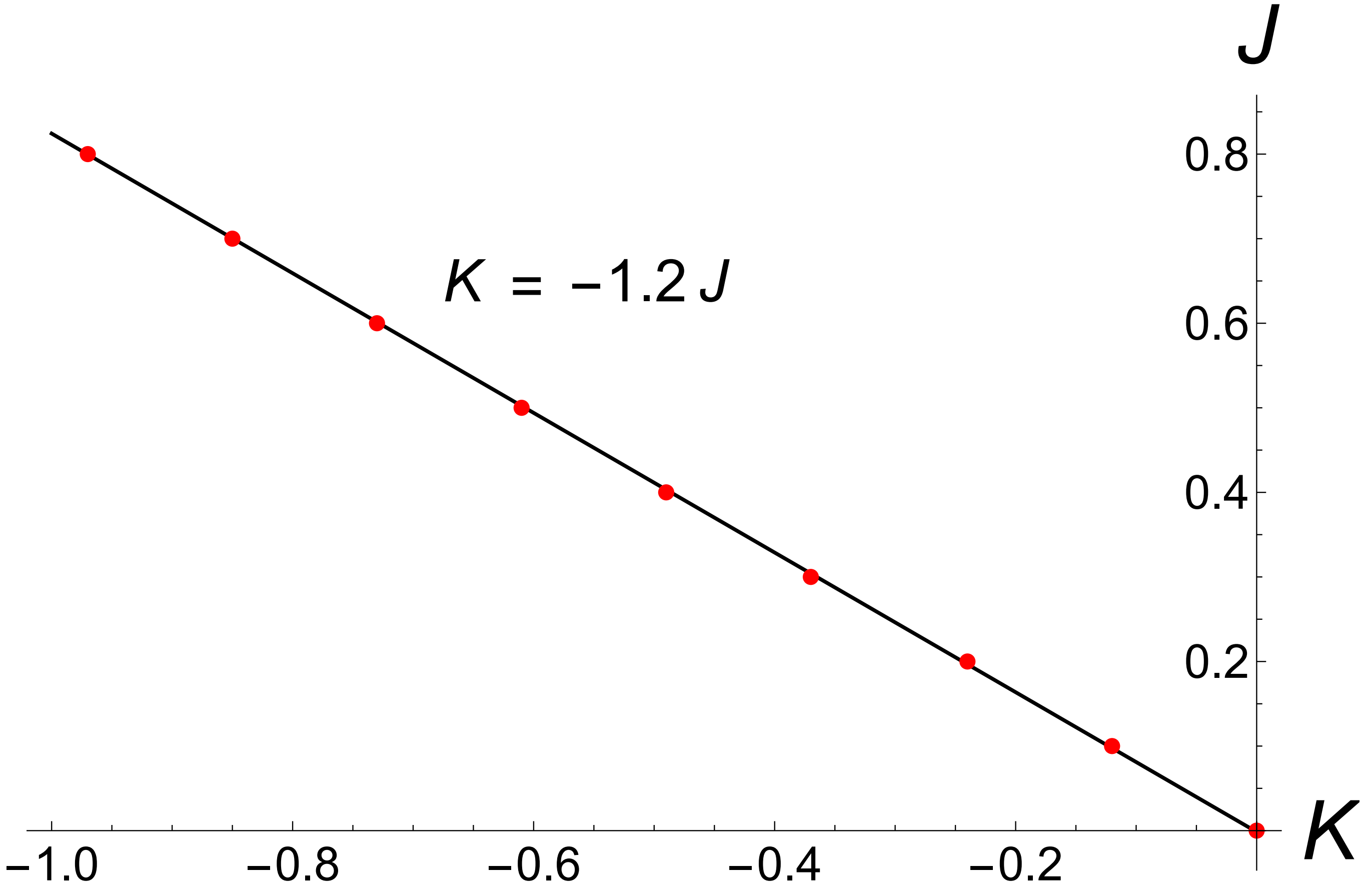

Supplementary Figure 6: **Critical coupling**. Critical coupling $K_c = K_1^*$ defined by supplementary equation (28) (red dots) versus J. The line of best fit is also shown.

SUPPLEMENTARY FIGURE 7

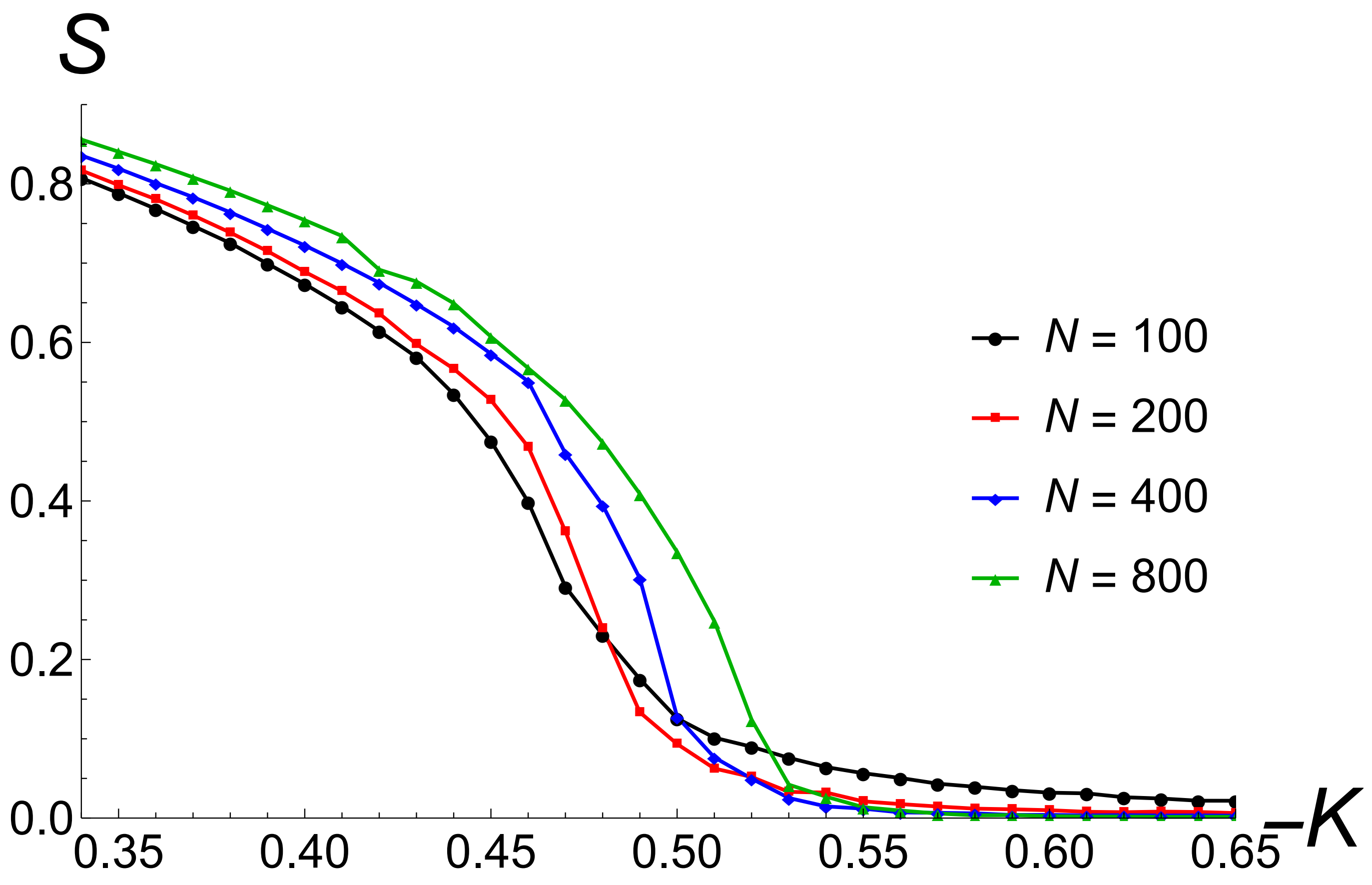

Supplementary Figure 7: **Finite size scaling of order parameter.** Behavior of order parameter $S = \max(S_+, S_-)$ versus K for $J = 0.5$ for increasing population sizes N. The critical coupling strength K_c at which S bifurcates from zero decreases with increasing N. Data were collected for $T = 1000$ time units with stepsize $dt = 0.01$. Each data point represents the average of 200 realizations.

SUPPLEMENTARY FIGURE 8

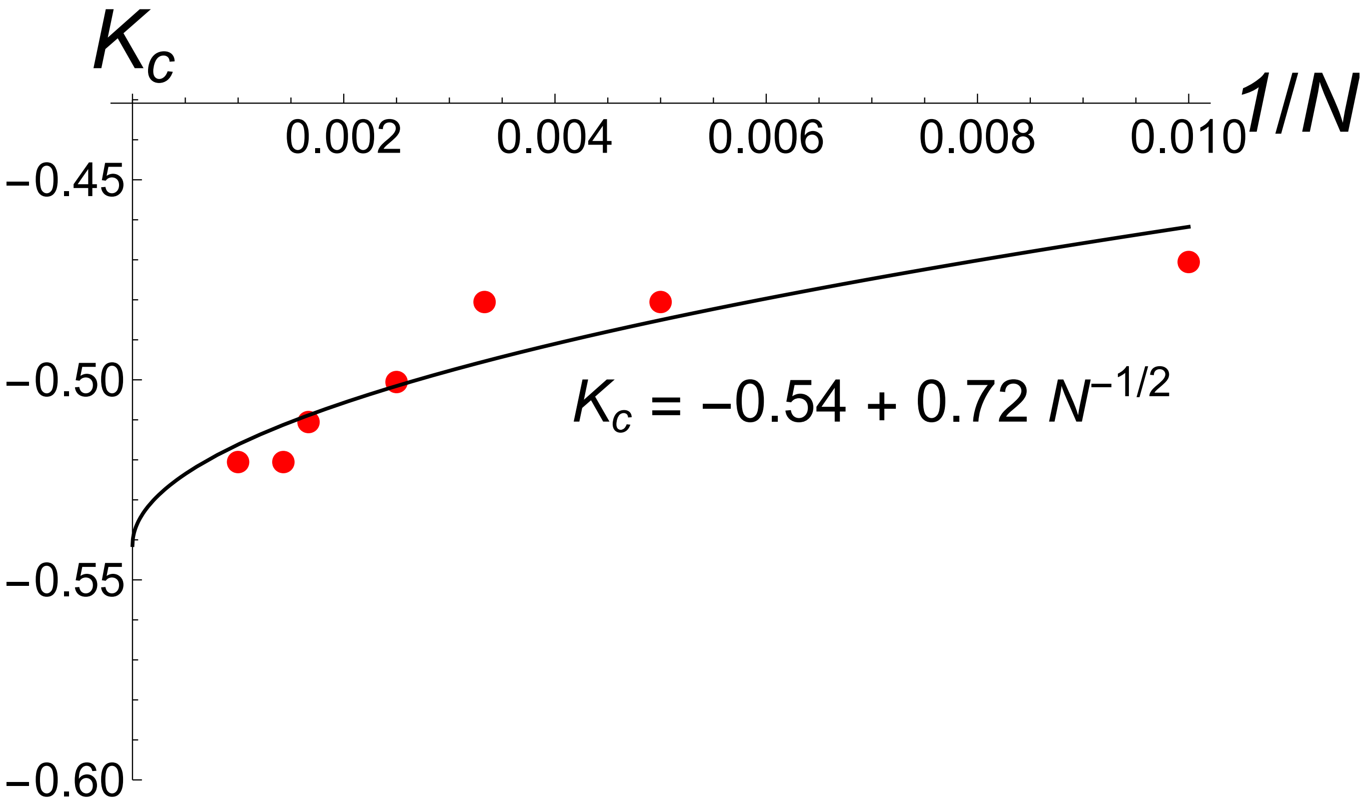

Supplementary Figure 8: **Finite size scaling of critical coupling strength** K_c**.** Behavior of the critical coupling strength K_c at which the order parameter S bifurcates from 0 for increasing population sizes. Red dots show simulation results. The black curve has form $K_c \approx a + bN^{-1/2}$, where the parameters of best fit were determined using Mathematica.

SUPPLEMENTARY FIGURE 9

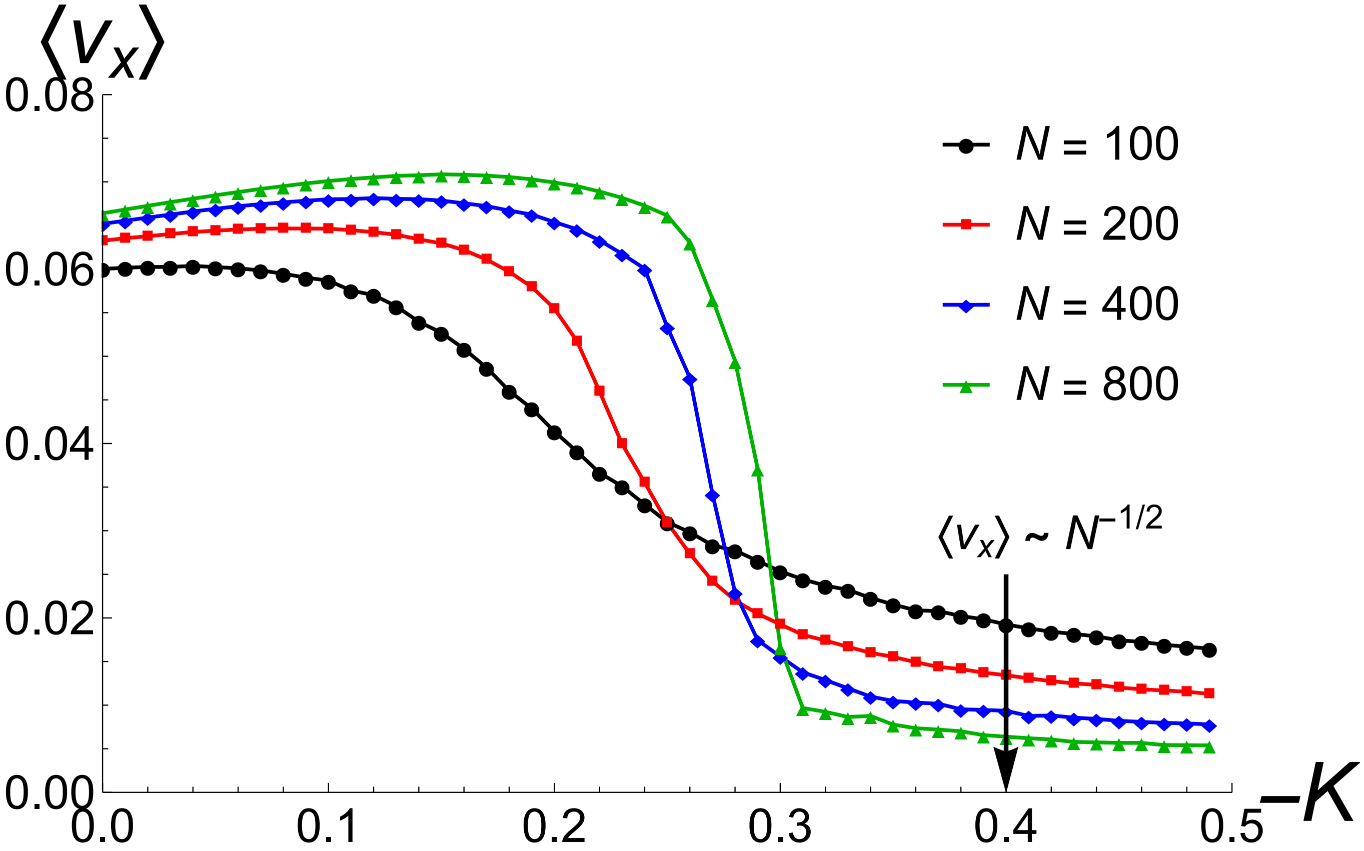

Supplementary Figure 9: Finite-size scaling of the asymptotic spatial velocity. Behavior of the asymptotic spatial velocity $\langle v_x \rangle$ versus K for $J = 0.5$ and $D_\theta = 0.06$ for increasing population sizes N. Simulations were run for 10^5 timesteps of size 0.01 using Heun's method. For sufficiently negative K, there is a drop in $\langle v_x \rangle$ indicating the transition to the active async state. In this state, the velocity decays as $\langle v_x \rangle \sim N^{-1/2}$.

SUPPLEMENTARY FIGURE 10

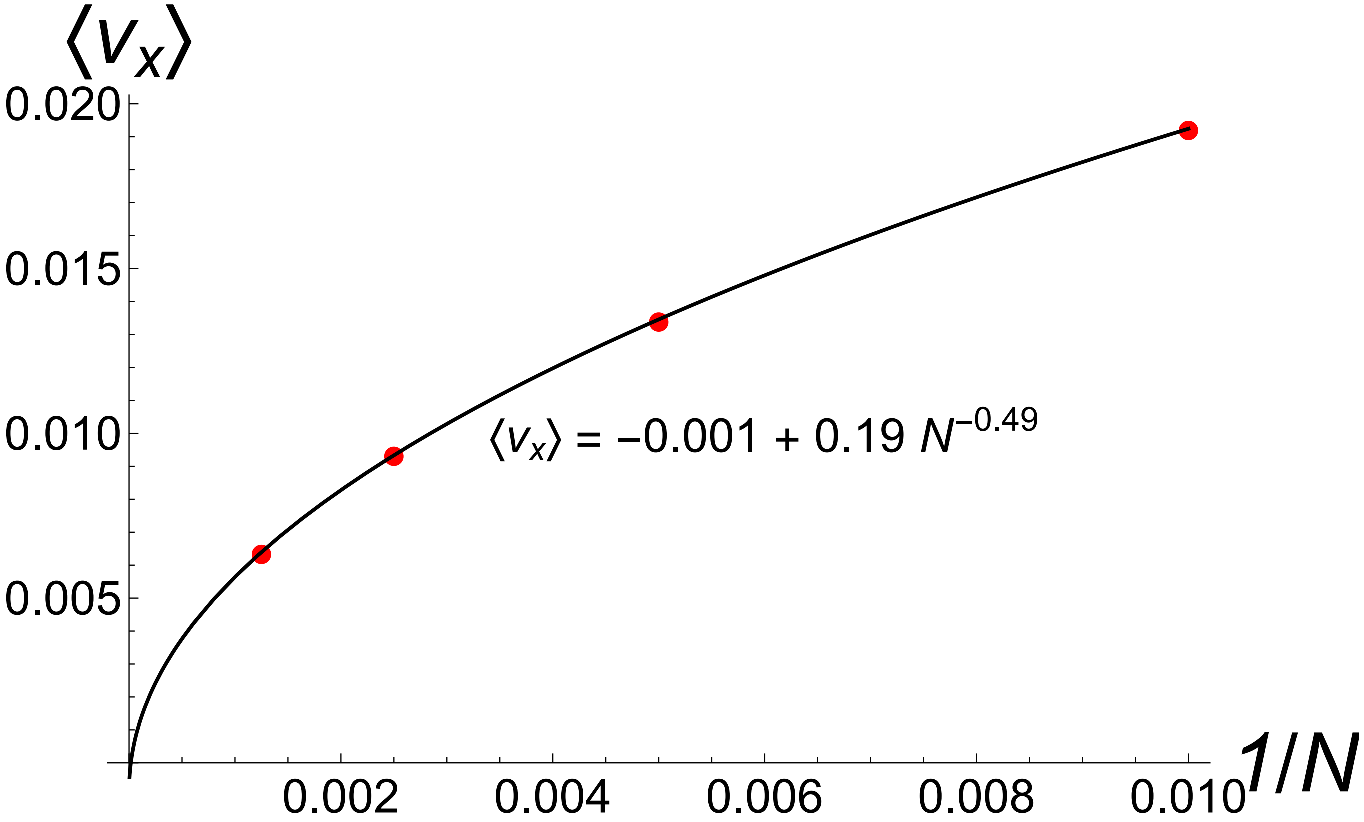

Supplementary Figure 10: **Finite-size scaling of the asymptotic spatial velocity.** Finite-size scaling of the asymptotic spatial velocity $\langle v_x \rangle$ versus population size N for $J = 0.5$, $D_\theta = 0.06$ and $K = -0.4$. Red dots shown simulation results for runs with 10^5 timesteps of size 0.01 using Heun's method. The solid black curve has form $a + bN^{-c}$, where a, b, c are the best fit parameters to the data. The $K_c \sim N^{-1/2}$ behavior is evident.

SUPPLEMENTARY FIGURE 11

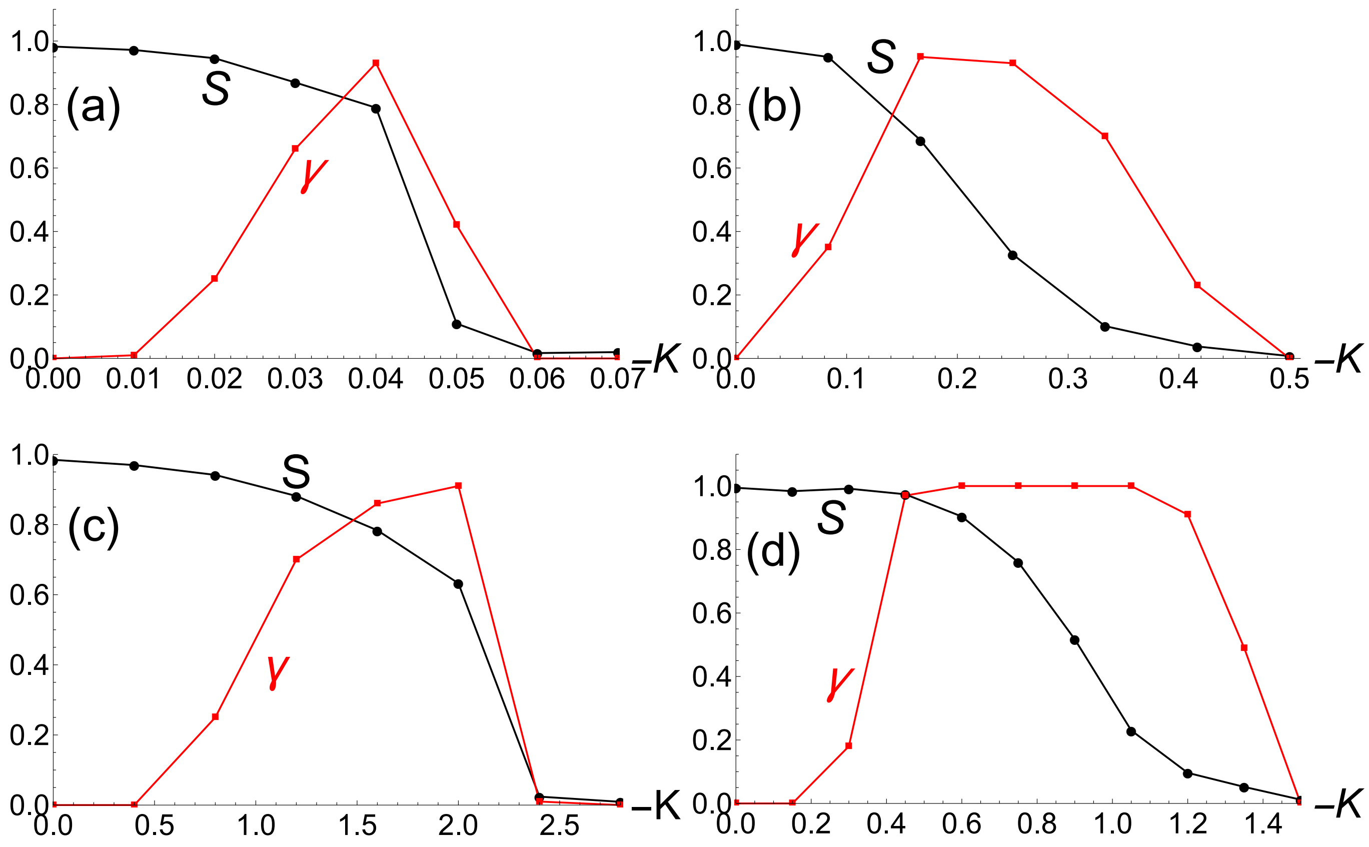

Supplementary Figure 11: **Genericity.** Behavior of the order parameters $S = \max(S_+, S_-)$ and γ for different choices of interactions functions. Unless otherwise stated, simulations were run for $N = 100$ swarmalators with $J = 1.0$ for $T = 1000$ time units with a stepsize of $dt = 0.5$ using python's 'odeint' solver. In all cases, the same qualitatively behavior was found, indicating the same states as the original model were realized. Panels (a) through (d) correspond to supplementary equations (34), through (37). In panel (c), $\nu = 0.1$ and in panel (d) $\nu = 3.0$. In panel (a) a longer time of $T = 2000$ was used.

SUPPLEMENTARY FIGURE 12

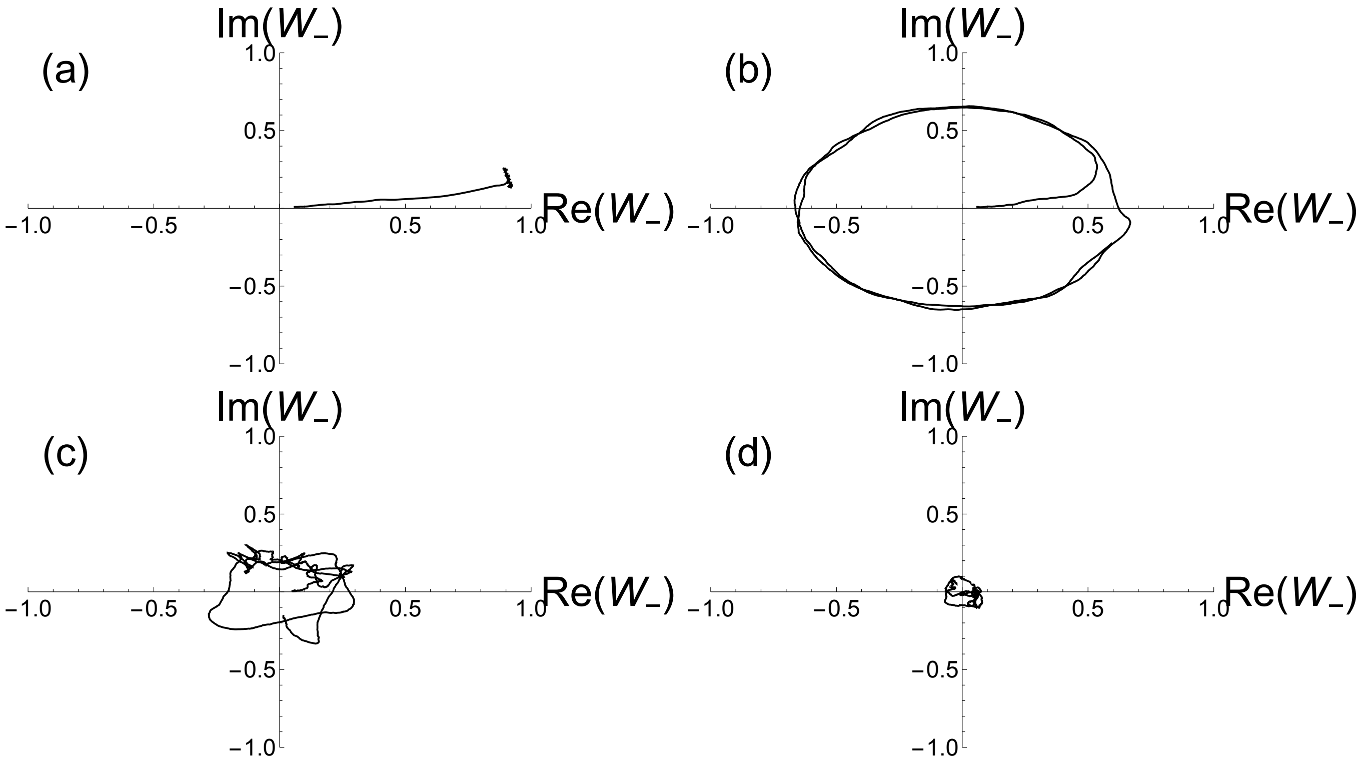

Supplementary Figure 12: **Order parameters for non-stationary phase waves.** Time series of the order parameters W_- in the complex plane for different values of K. In all panels, simulations were run for $N = 1000$ swarmalators with $(dt, T) = (0.5, 500)$ and $J = 0.5$. (a) $K = -0.3$. Both the phase and amplitude of W_- approach constant values, indicating the active phase wave state. (b) $K = -0.4$. W_- rotates with constant velocity and amplitude, indicating the non-stationary phase wave state. (c) $K = -0.5$. Both the amplitude and phase of W_- oscillate irregularly, indicating another version of the non-stationary phase wave state. (d) $K = -0.8$. Both the amplitude and phase of W_- are zero (up to finite effects), indicating the static async state.

SUPPLEMENTARY FIGURE 13

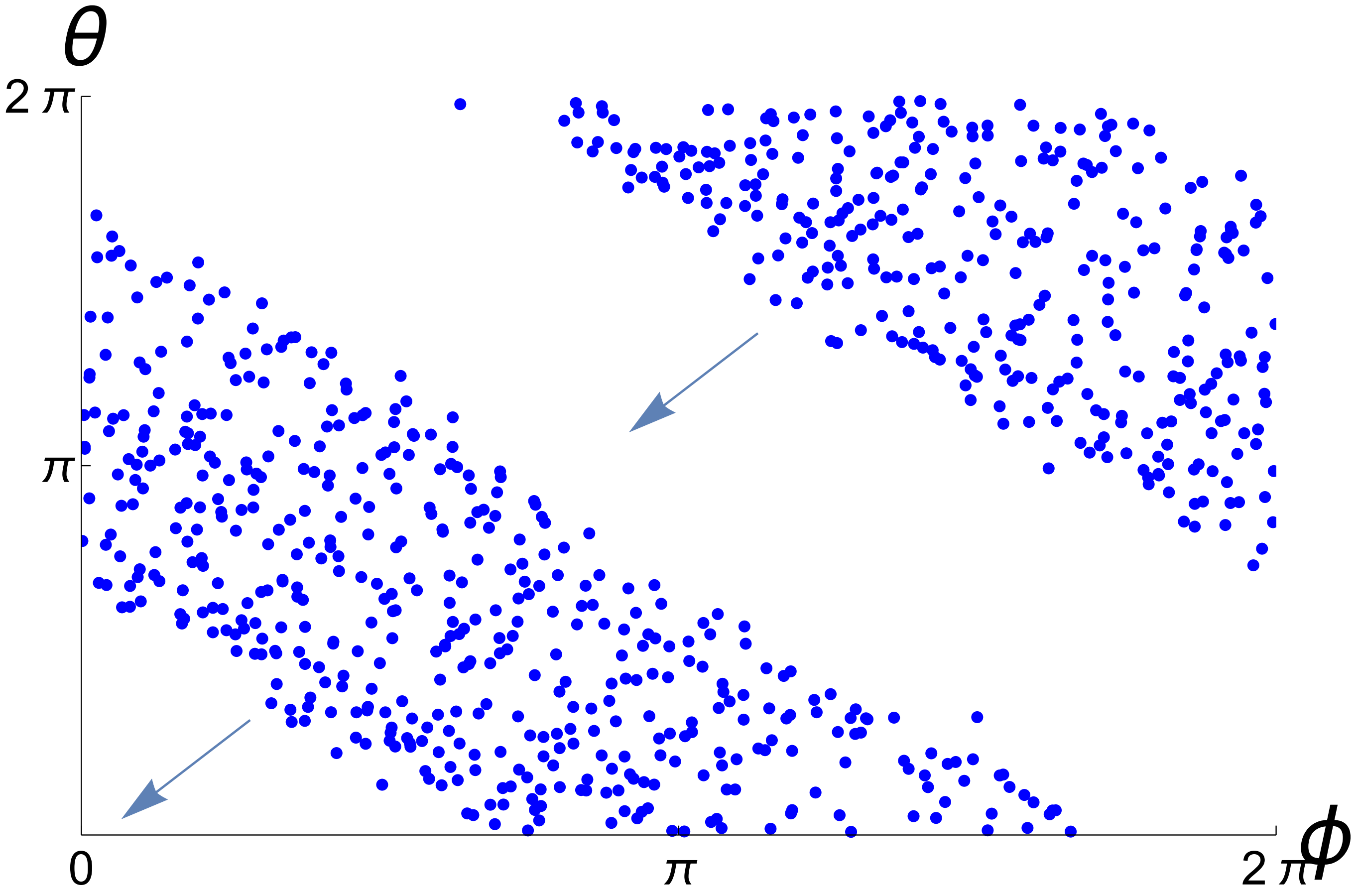

Supplementary Figure 13: **Non-stationary phase waves**. Scatter plots of the swarmalators' spatial angles ϕ and phases θ in the non-stationary phase wave state. Simulations were performed for $N = 800$ swarmalators with $dt = 0.01$ and $(J, K) = (0.9, -0.11)$ using python's ode solver 'odeint'. As illustrated by the arrows, the offset of the correlation between ϕ and θ changes uniformly between 0 and 2π.

SUPPLEMENTARY FIGURE 14

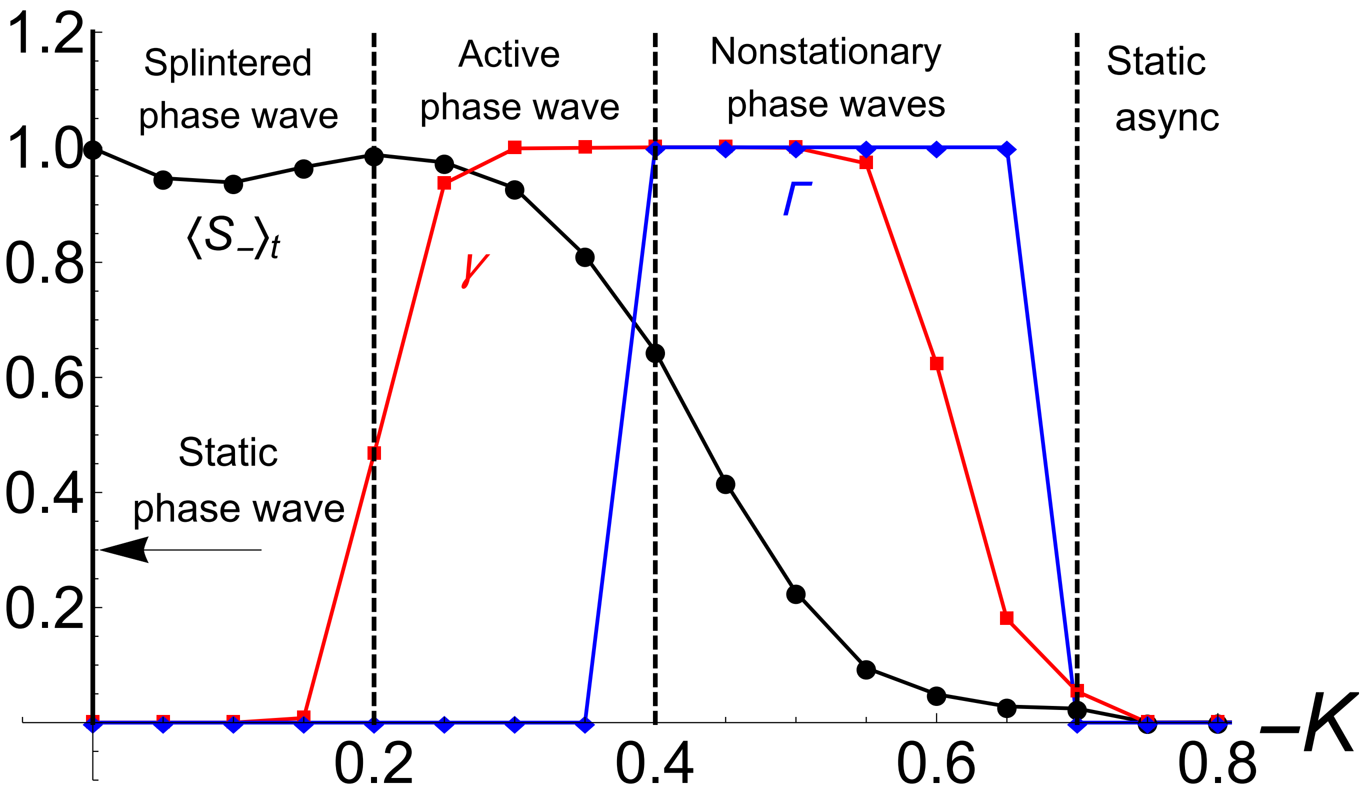

Supplementary Figure 14: **Order parameters for linear attraction kernel.** Order parameters for $N = 1000$ swarmalators with $(dt, T) = (0.5, 1000)$ and $J = 0.5$ and $\mathbf{I}_{\rm att}(\mathbf{x}) = \mathbf{x}$. The first 50% of data were discarded as transients. Angled brackets $\langle . \rangle$ denote time average. Plots are qualitatively similar for other values of J.

SUPPLEMENTARY FIGURE 15

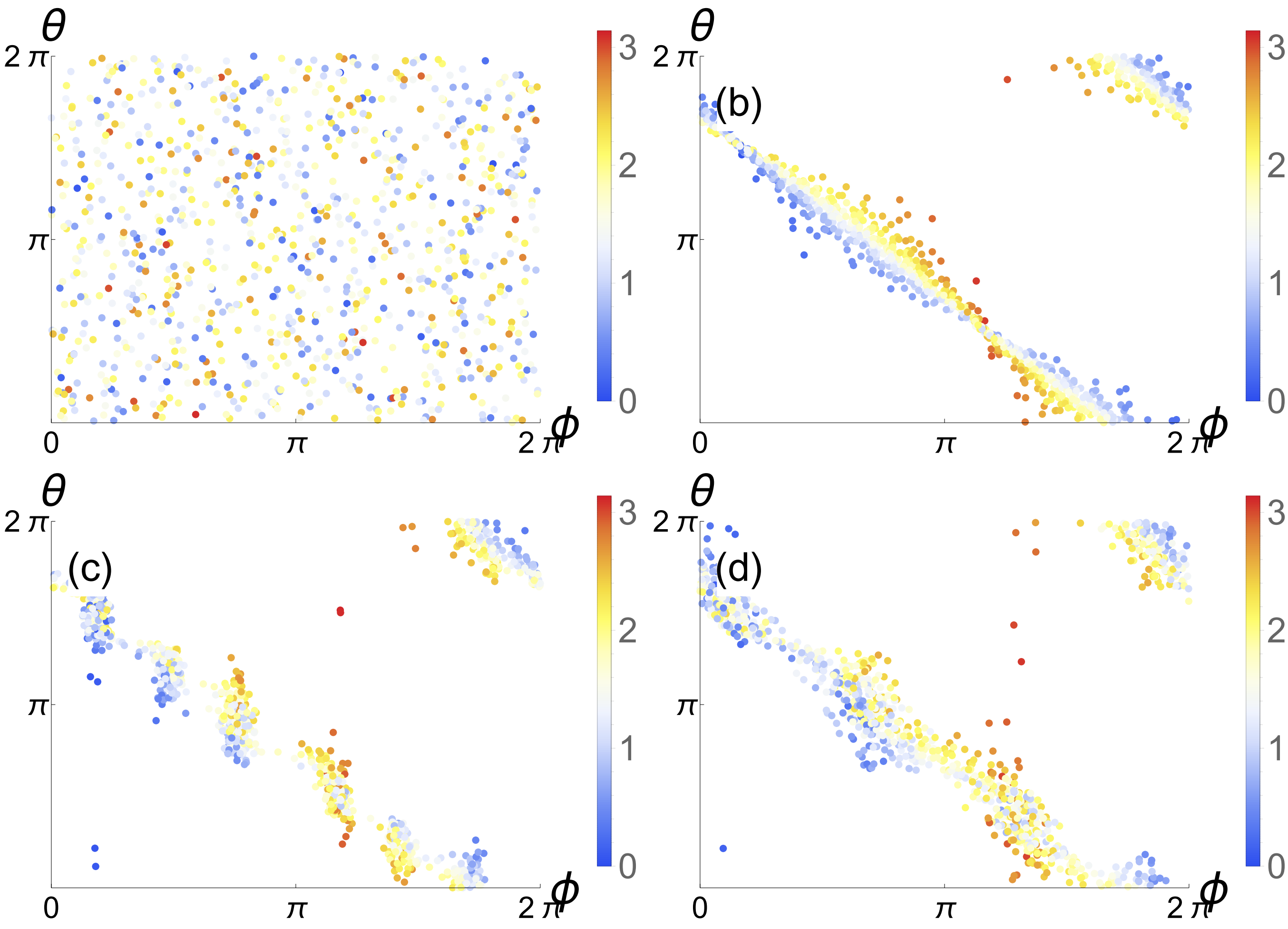

Supplementary Figure 15: **Scatter plots for swarmalators in 3D**. Scatter plots of four states in the $(\phi.\theta)$ plane, where the swarmalators are colored according to their polar angle α. Data were collected for $N = 1000$ swarmalators for 5×10^5 timesteps of width $dt = 0.001$ using Heun's method. The first 50% of data were discarded as transients. Parameter values were $(J, \sigma, D_\theta, D_x, D_y, D_z) = (0.5, 0, 0, 0, 0, 0)$. (a) Static async state for $K = -1$. (b) Static phase wave for $K = 0$. (c) Splintered phase wave for $K = -0.05$. (d) Active phase wave state for $K = -0.6$.

SUPPLEMENTARY FIGURE 16

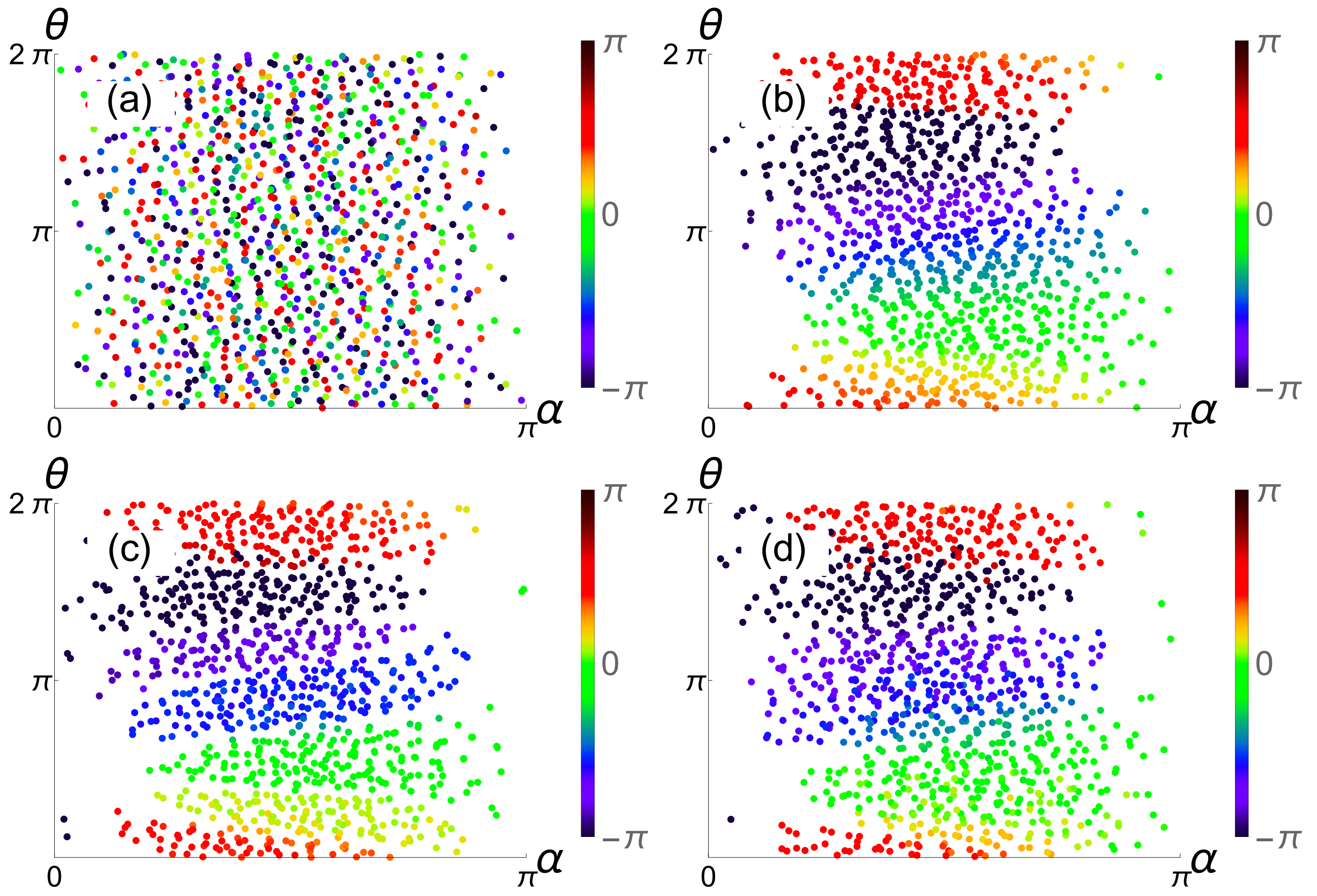

Supplementary Figure 16: **Scatter plots for swarmalators in 3D**. Scatter plots of four states in the $(\alpha.\theta)$ plane, where the swarmalators are colored according to their polar azimuthal angle ϕ. Parameter and simulations details are the same as for Figure 15.

SUPPLEMENTARY FIGURE 17

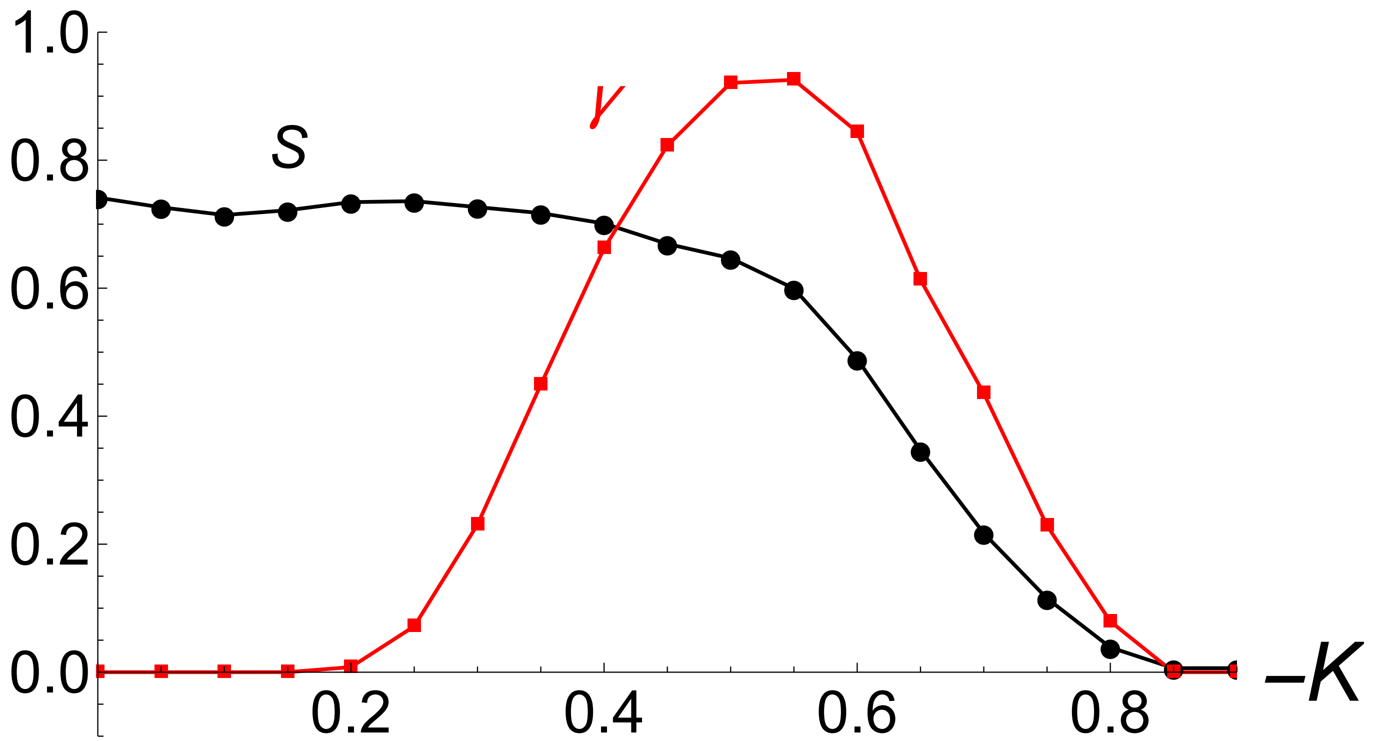

Supplementary Figure 17: **Order parameters for swarmalators in 3D.** Behavior of 3D versions of the order parameters $S_{\pm} = |N^{-1} \sum_j e^{i(\phi_j \pm \theta_j)}|$ and γ when $J = 0.5$. Both $S = \max(S_+, S_-)$ and γ have the same qualitative behavior as their 2D counterparts. Simulations have been run for $N = 500$ swarmalators using Heun's method for 5×10^5 time steps of width 0.001. One hundred realizations were computed for each value of K, the average of which is plotted.

SUPPLEMENTARY FIGURE 18

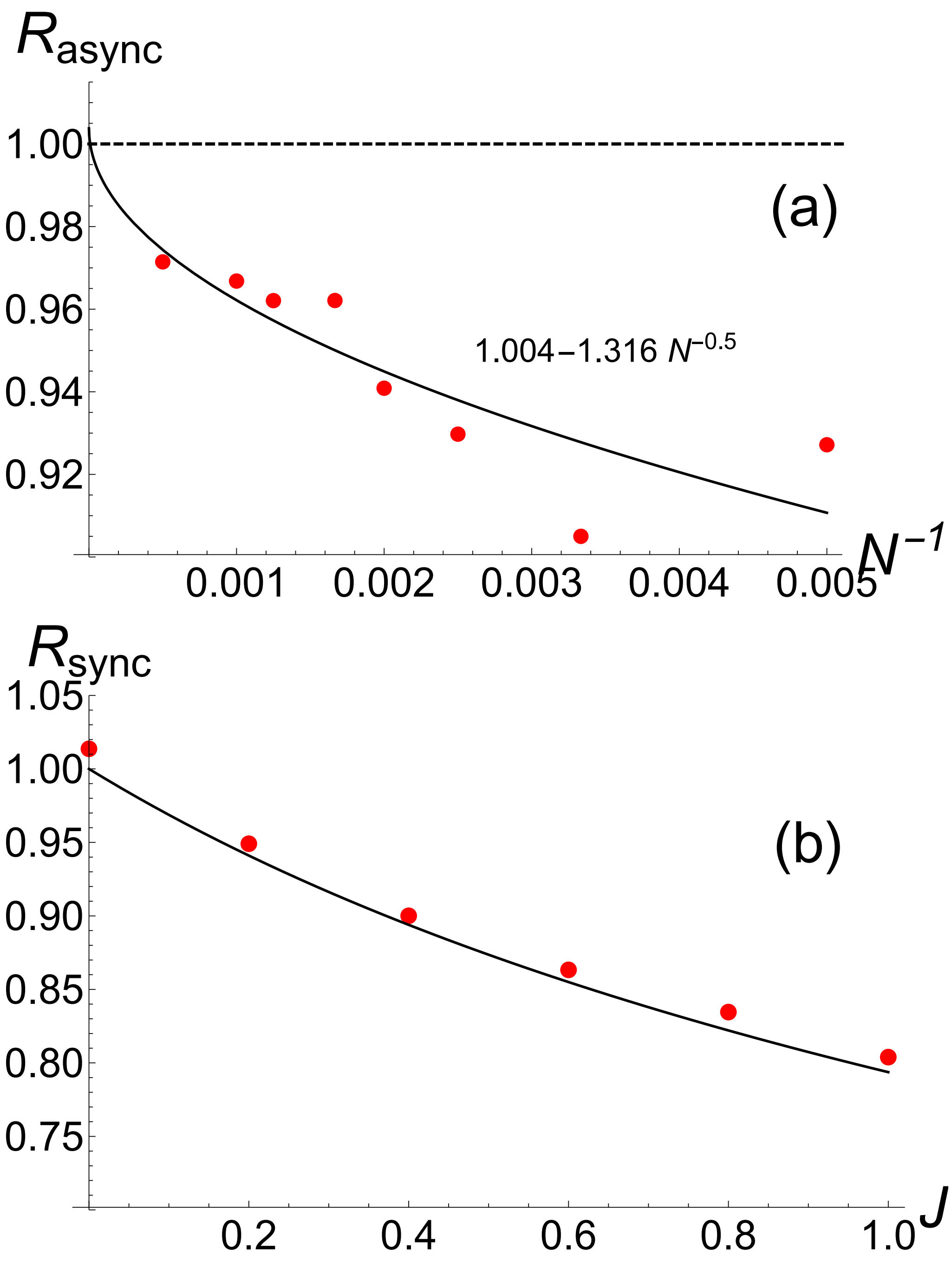

Supplementary Figure 18: **Testing predictions for the support radii in 3D.** (a) Finite-size scaling of the radius of static async state for $J = K = 0$. Red dots show simulation results, the dotted black line shows theoretical prediction $R_{\text{async}} = 1$, and the solid black line shows the curve $a + bN^{-c}$ where the parameters of best fit have been found using Mathematica. As can be seen, the data approach the theoretical prediction as $N^{-1} \to 0$, as confirmed by the best fit parameter $a = 0.998$. (b) Radius of static sync state as a function of J for $K = 1$. The solid black line shows the theoretical prediction $R_{\text{sync}} = (1 + J)^{-1/3}$. For each each value of J, the radius $R_{\text{sync}}(N)$ was calculated for $N = 100, 200, 500, 1000$ swarmalators, from which the $N \to \infty$ limit was found by fitting the data to a curve $a + bN^{-0.5}$, and identifying $a = R_{\text{sync}}$. In both panels, simulations were run for $T = 500$ time units with a stepsize of 0.5 using python's solver 'odeint'.

SUPPLEMENTARY FIGURE 19

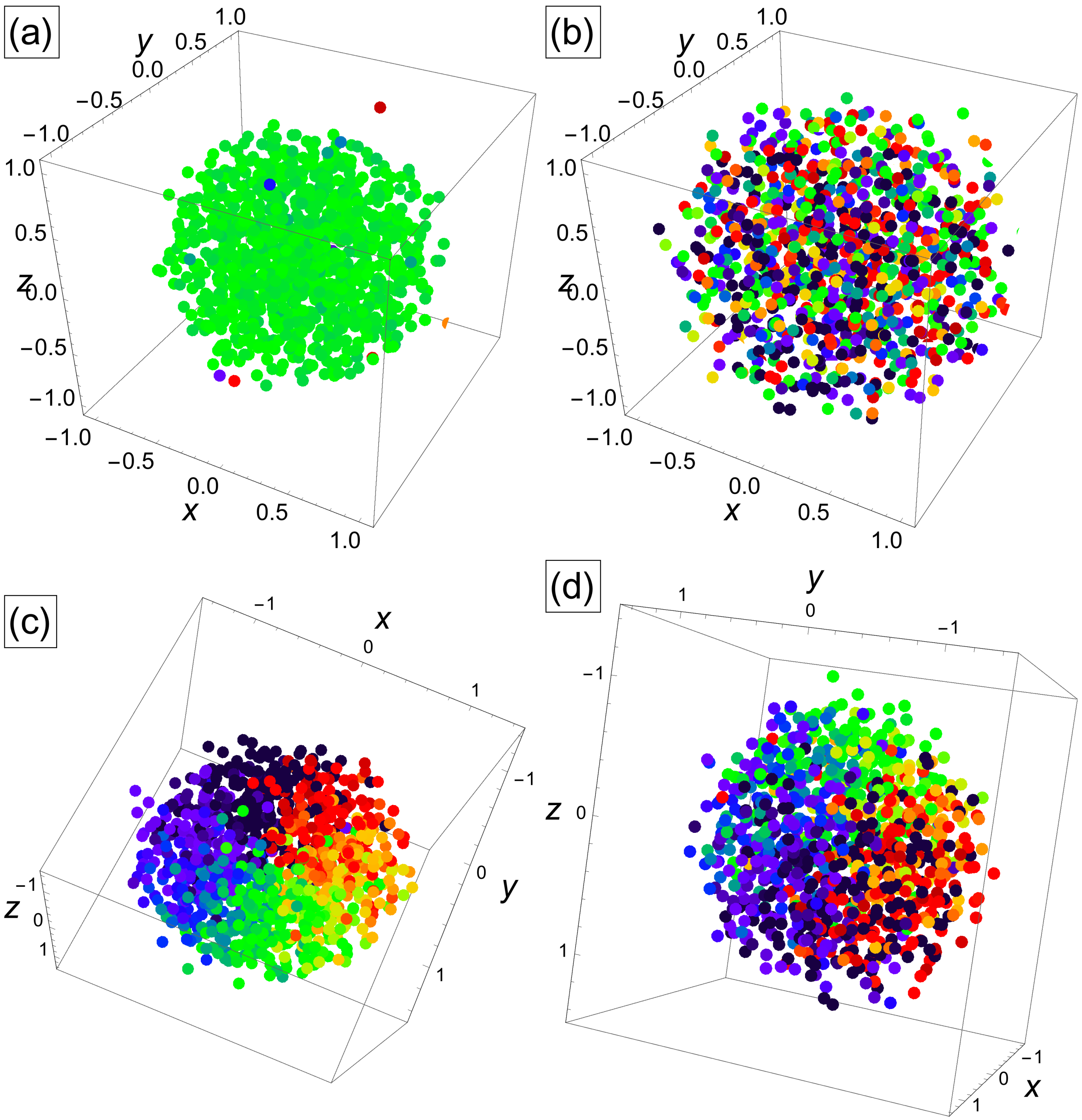

Supplementary Figure 19: **Scatter plots for swarmalators in 3D**. Scatter plots of four states in the (x, y, z) plane, where the swarmalators are colored according to their phase. Data were collected for $N = 1000$ swarmalators for 5×10^5 timesteps of width $dt = 0.001$ using Heun's method. Parameter values were $(J, \sigma, D_\theta, D_x, D_y, D_z) = (0.5, 0.01, 0.01, 0.01, 0.01, 0.01)$. (a) Active sync state for $K = 1$. (b) Active async state $K = -1$. (c) Active phase wave state for $K = 0$. (d) Active phase wave state for $K = -0.6$.

SUPPLEMENTARY FIGURE 20

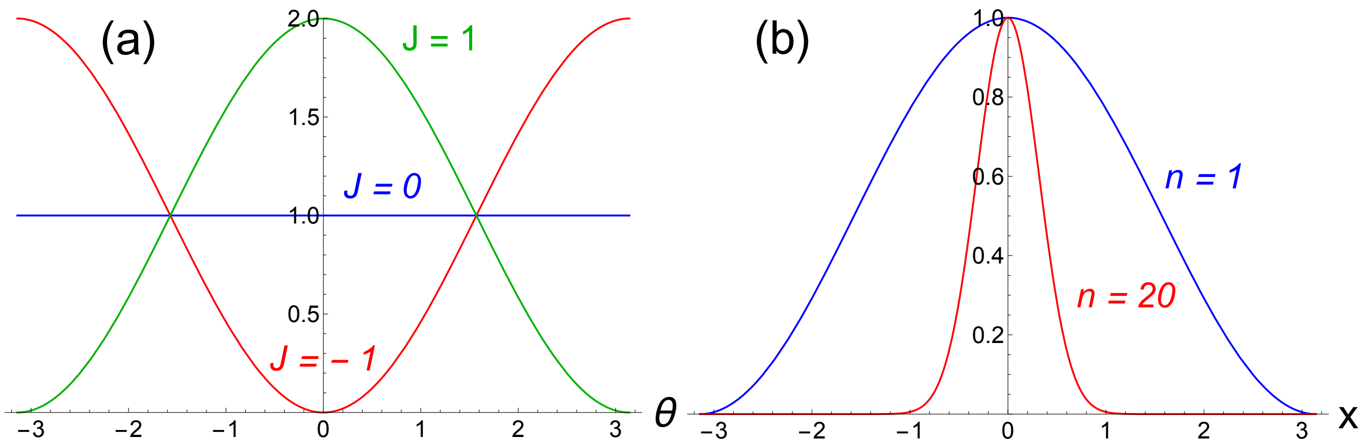

Supplementary Figure 20: **Pulse functions for swarmalators in 1D**. (a) $F(\theta) = 1 + J\cos(\theta)$ for $J = 0, -1, 1$. (b) The pulse function $2^{-n}(1 + \cos x)^n$ for $n = 1, 20$, showing a decrease in width for increasing n.

SUPPLEMENTARY FIGURE 21

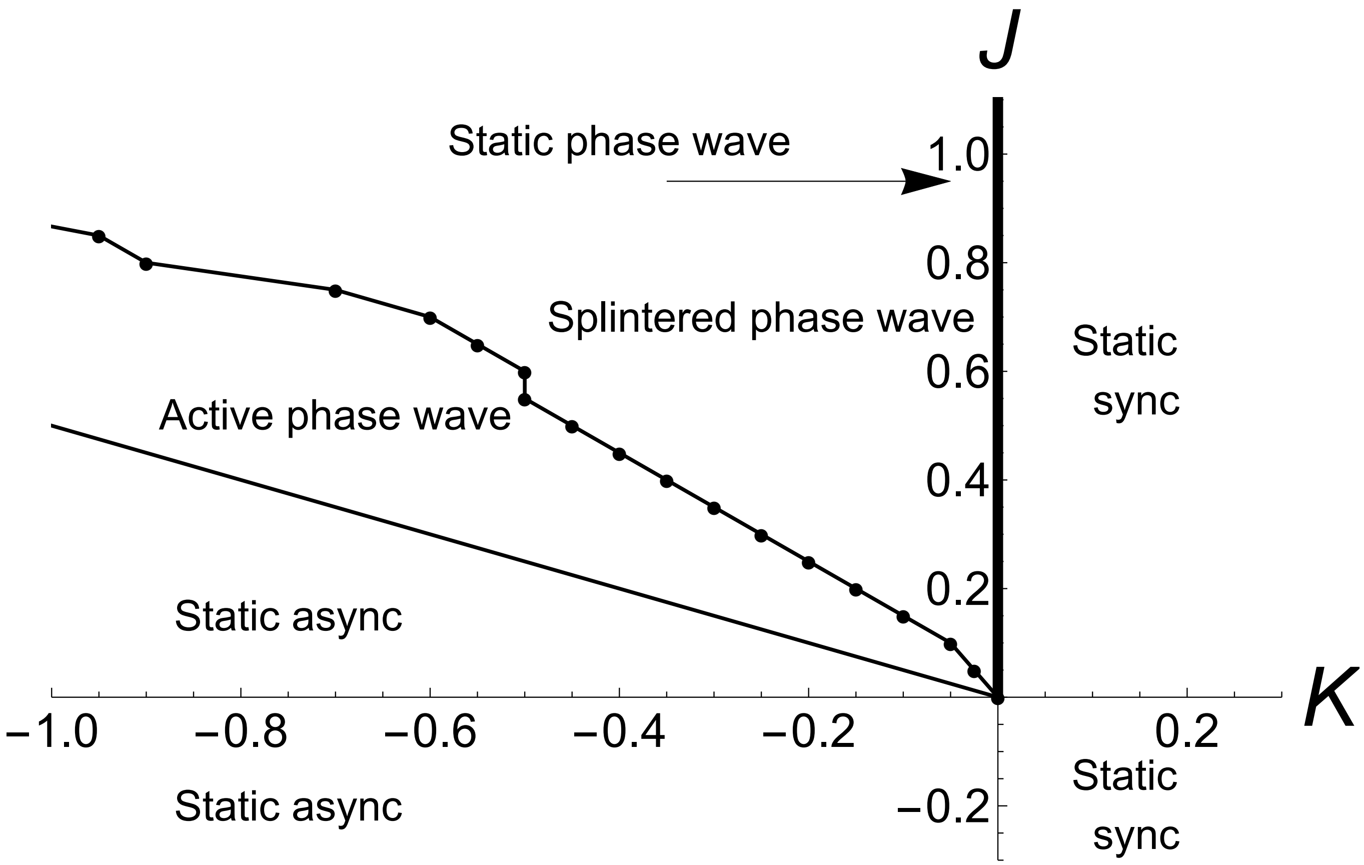

Supplementary Figure 21: **Phase diagram for swarmalators in 1D**. The line separating the static async and active phase wave states was calculated analytically and is given by supplementary equation (43). The line separating the active phase wave and splintered phase wave states was calculated numerically, where black dots show simulation results.

SUPPLEMENTARY FIGURE 22

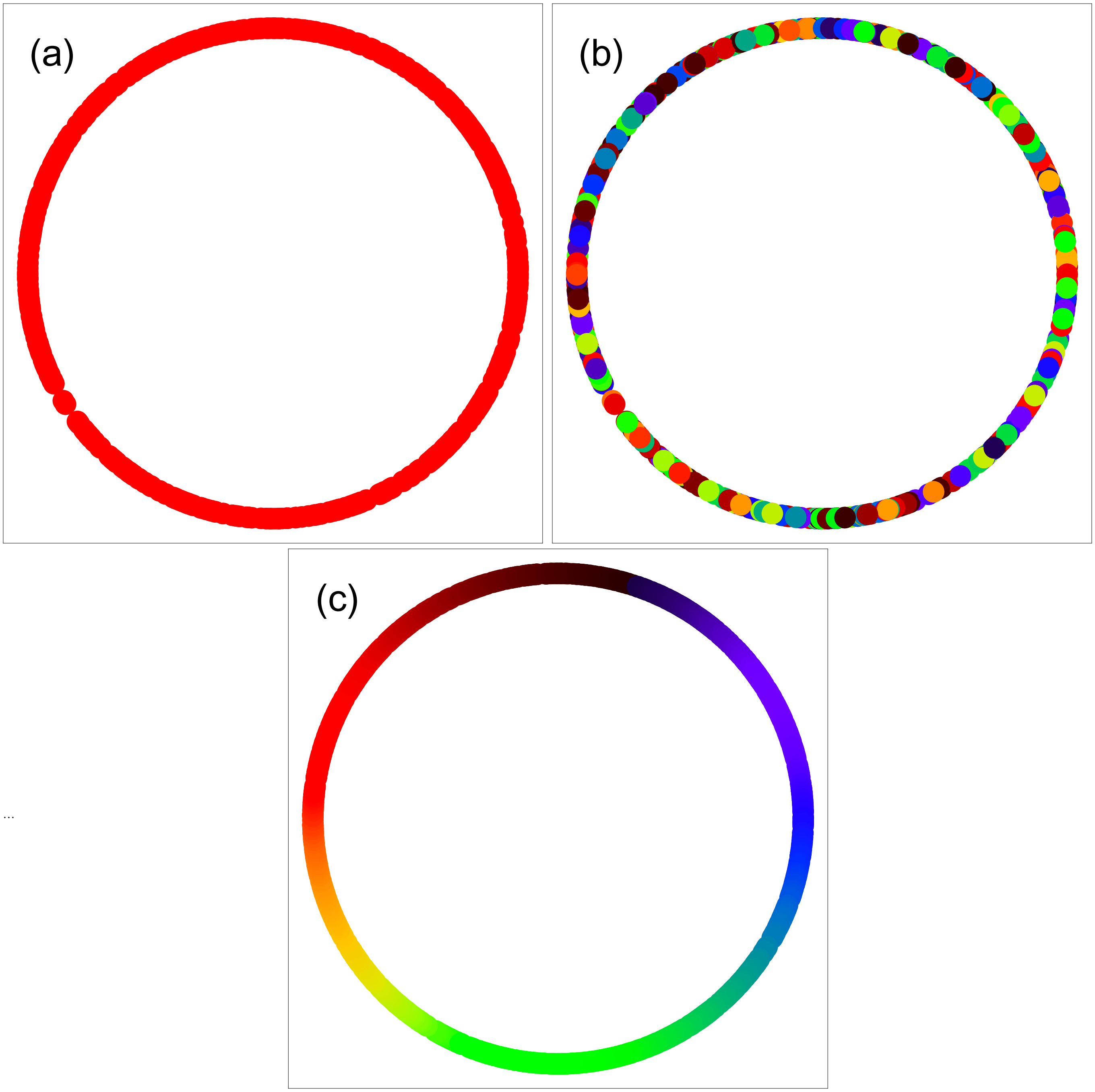

...

Supplementary Figure 22: **Scatter plots for swarmalators in 1D**. Three steady states for $N = 1000$ swarmalators with a time step of 0.1. Swarmalators' initial phases and positions were drawn uniformly at random from $[-\pi, \pi]$. Swarmalators are positioned on the unit circle and are colored according to their phase. (a) Static sync state for $(J, K) = (1, 1)$. (b) Static async state for $(J, K) = (1, -1.2)$. (c) Static phase wave state $(J, K) = (1, 0)$.

SUPPLEMENTARY FIGURE 23

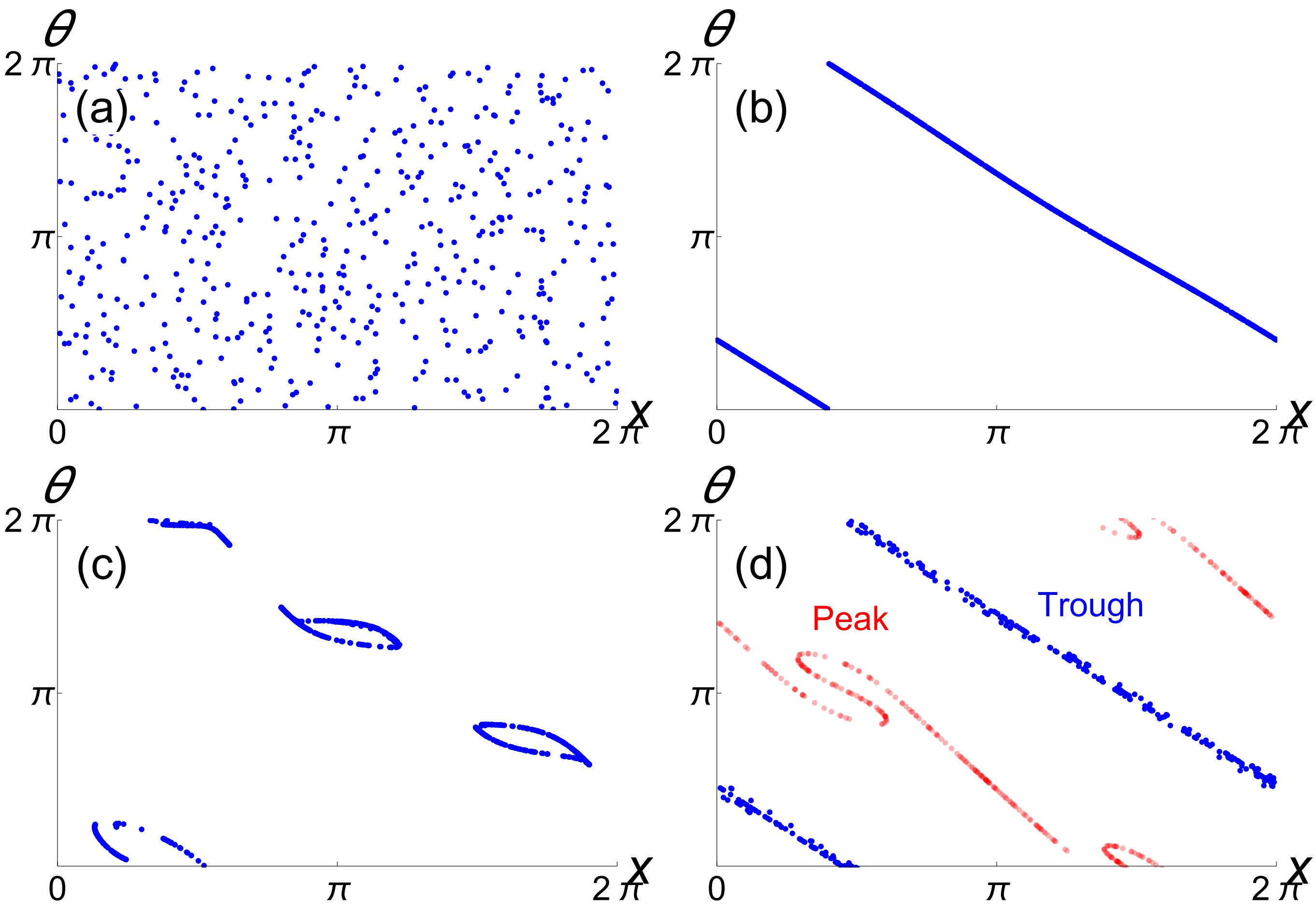

Supplementary Figure 23: **Stationary states in 1D**. Distributions of different states in (x, θ) space. In all panels, simulations were run with $N = 1000$ swarmalators for 500 time units with a step of $dt = 0.1$. (a) Static async state for $(J, K) = (1, 1)$. (b) Static phase wave state $(J, K) = (1, 0)$. (c) Splintered phase wave state $(J, K) = (1, -0.25)$. Note the clusters gently pulsate. (d) Active phase wave state $(J, K) = (0.1, -1)$. Blue dots, distribution during a trough (when the mean population speed $\langle v \rangle$ is minimum). Red dots, distribution during a peak (when $\langle v \rangle$ is a maximum). The peak distribution has been shifted by $-\pi$ in the x direction for clarity.

SUPPLEMENTARY FIGURE 24

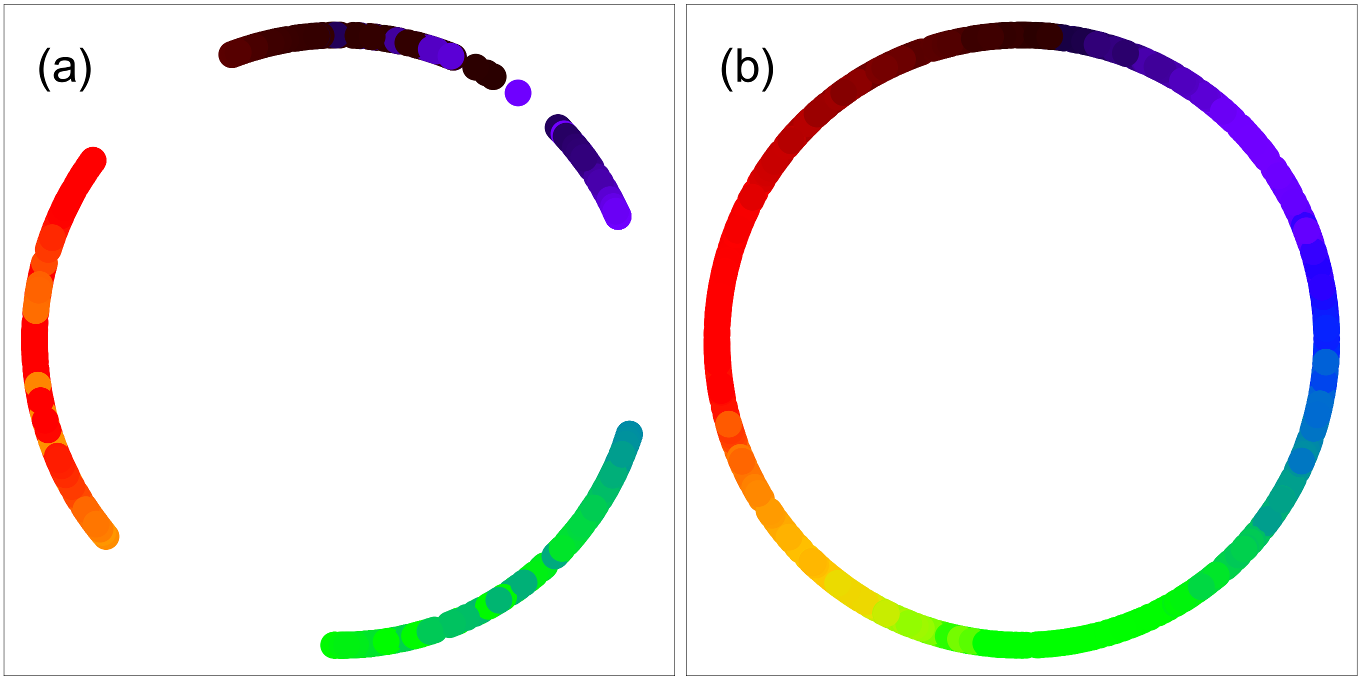

Supplementary Figure 24: **Non-stationary states in 1D**. Two non-steady states for $N = 1000$ swarmalators with a time step of 0.1. In all cases, swarmalators' initial phases and positions were drawn uniformly at random from $[-\pi, \pi]$. (a) Splintered phase wave for $(J, K) = (1, -0.25)$ and $n = 3$ (as opposed to $n = 1$). (b) Active phase wave for $(J, K) = (1, -1.5)$.

SUPPLEMENTARY FIGURE 25

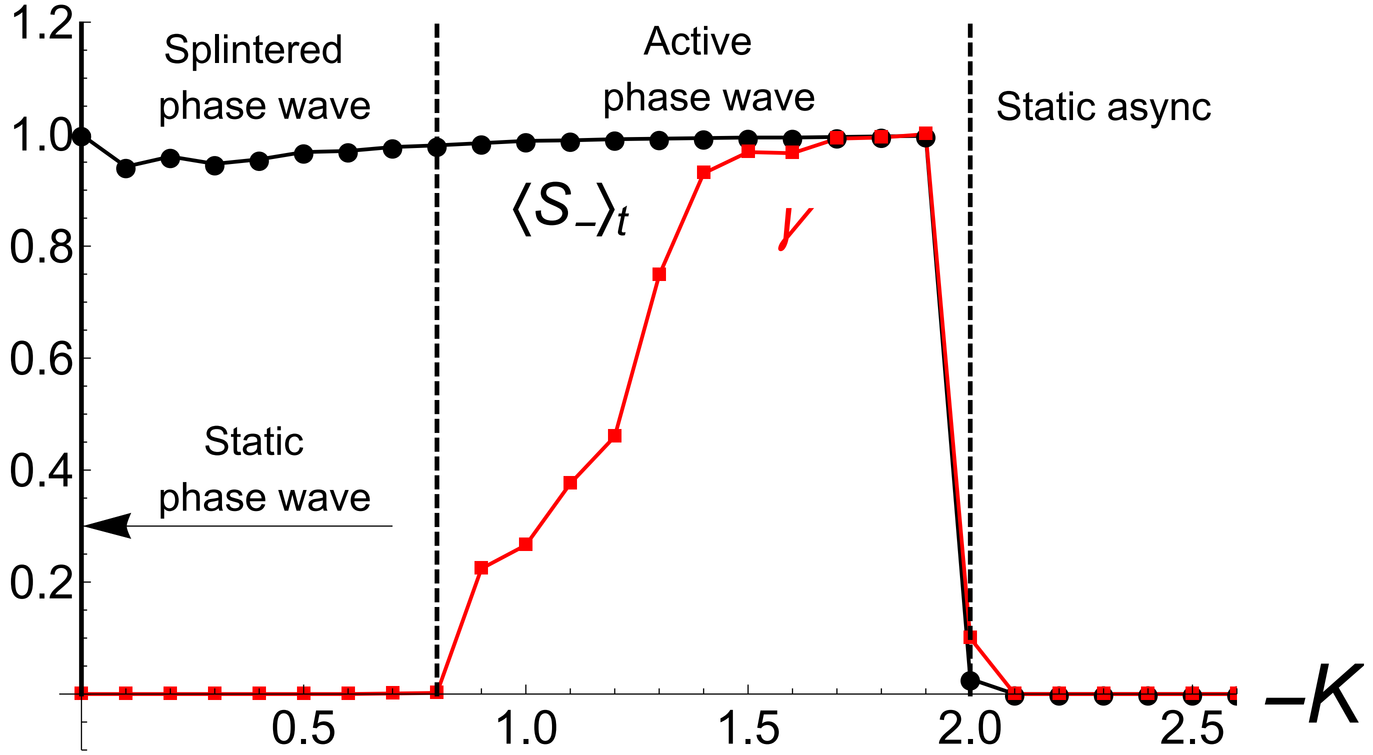

Supplementary Figure 25: **Order parameters for swarmalators in 1D.** Time average of order parameter S, and γ for $J = 1.0$ and $N = 500$ swarmalators. Data was collected for $T = 3000$ time units, of which the first 50% of data were discarded as transients. As can be seen γ bifurcates from 0 at $K \approx -0.8$, signaling the transition from the splintered, to the active, phase wave states. At $K_c = -2J = -2$, S_- bifurcates to zero indicating the transition to the static async state, as predicted by supplementary equation (43).

SUPPLEMENTARY FIGURE 26

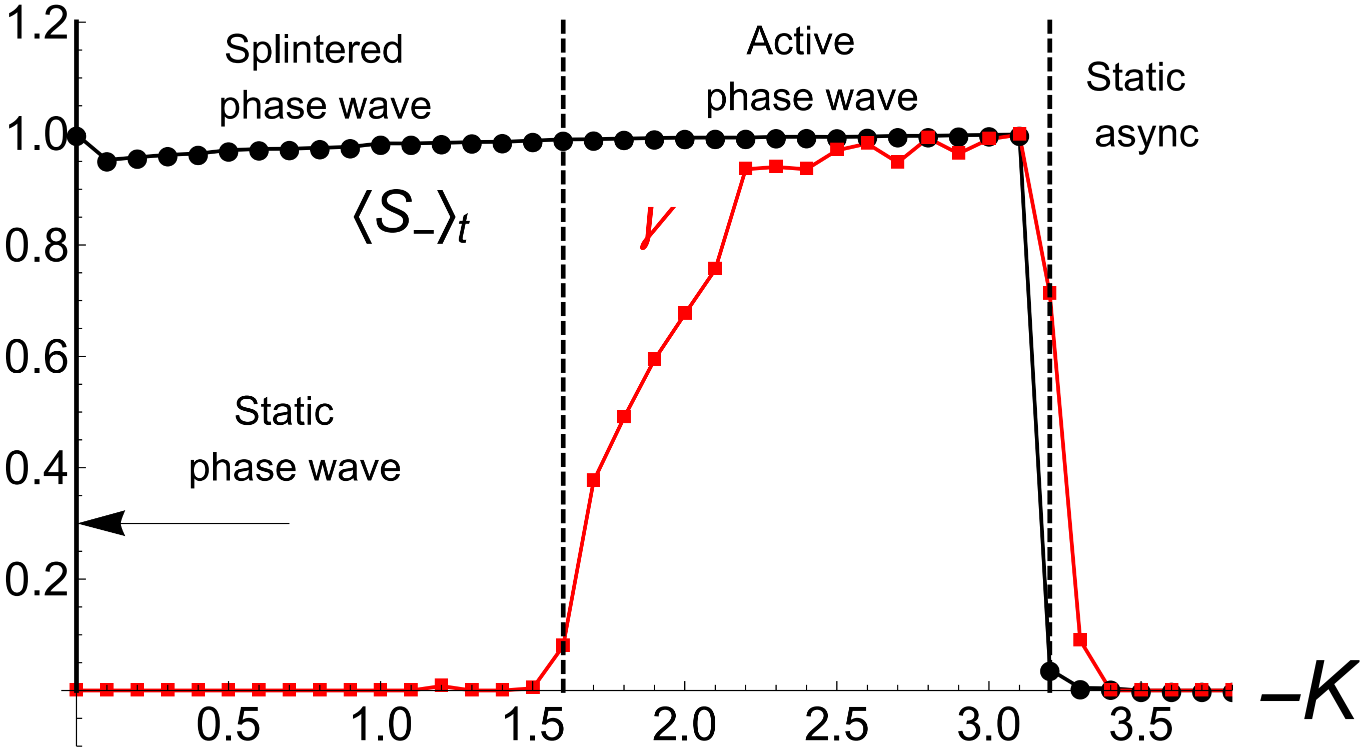

Supplementary Figure 26: **Order paramters for swarmalators in 1D with different interaction functions**. Time averaged of order parameter S, and γ for $J = 1.0$ and $N = 500$ swarmalators for $G(x)$ given by supplementary equation (46). Data was collected for $T = 3000$ time units, of which the first 50% of data were discarded as transients. Notice the order parameters for this choice of $G(x)$ have the same qualitative behavior as that for the original model defined by supplementary equations (41) and (42) as seen in Supplmental Fig. 25.

SUPPLEMENTARY NOTE 1

Static async state with unit vector attraction kernel. We here semi-analytically derive the density and radius of the static async state when $I_{\rm att}(\mathbf{x}) = \mathbf{x}/|\mathbf{x}|$. We use the techniques outlined in the Methods section of the main text. This amounts to solving the pair of equations

$$\underline{v} \equiv \underline{0}, \tag{1}$$
$$\nabla.\underline{v} \equiv 0, \tag{2}$$

where the velocity is

$$\mathbf{v_x}(\mathbf{x},\theta,t) = \int \left(\frac{\tilde{\mathbf{x}}-\mathbf{x}}{|\tilde{\mathbf{x}}-\mathbf{x}|}\Big(1 + J\cos(\tilde{\theta}-\theta)\Big) - \frac{\tilde{\mathbf{x}}-\mathbf{x}}{|\tilde{\mathbf{x}}-\mathbf{x}|^2} \right) \rho(\tilde{\mathbf{x}},\, \tilde{\theta}, t)\, d\tilde{\mathbf{x}}\, d\tilde{\theta} \tag{3}$$

$$v_\theta(\mathbf{x},\theta,t) = \int \frac{\sin(\tilde{\theta}-\theta)}{|\tilde{\mathbf{x}}-\mathbf{x}|} \rho(\tilde{\mathbf{x}},\tilde{\theta},t) d\tilde{\mathbf{x}}\, d\tilde{\theta}. \tag{4}$$

And the density ansatz is

$$\rho(r,\phi,\theta,t) = \frac{1}{4\pi^2} g(r), \qquad 0 \le r \le R. \tag{5}$$

After calculations we get the following pair of simultaneous equations for the unknown radial density $g(r)$ and radius R,

$$\int_0^R \left[(s-r)\mathcal{K}\left(\frac{4rs}{(r+s)^2}\right) + (r+s)\mathcal{E}\left(\frac{4rs}{(r+s)^2}\right) + \frac{\pi^2}{2J}(r-s) \right] \frac{2Js}{r} g(s)\, ds = 0 \tag{6}$$

$$g(r) = \frac{2(1+J)}{\pi} \int_0^R \mathcal{K}\left(\frac{4sr}{(r+s)^2}\right) \frac{g(s)}{s+r} s\, ds, \tag{7}$$

where $\mathcal{K}, \mathcal{E}$ are the complete elliptic integrals of the first and second kinds, and R is the radius of the disk in the (x,y) plane which must be determined. We were unable to solve these equations for $g_1(r)$ and R.

Supplementary equation (7) is however easily solved numerically by discretizing the interval $[0,R]$, which leads to

$$g_i = M_{ij} g_j, \tag{8}$$
$$M_{ij} = \frac{2(1+J)}{\pi} \mathcal{K}\left(\frac{4r_j r_i}{(r_i+r_j)^2}\right) \frac{r_j}{r_j+r_i} w_j. \tag{9}$$

where $i = 1, \dots N_{grid}$, $r_i = i * h = i * (R/N_{grid})$, $g_i = g(r_i)$ and w_j are gaussian quadrature weights. We see that g_i is simply an eigenvector of the matrix M_{ij} with eigenvalue 1. This lets us find the unknown radius R by computing the eigenvalues of M_{ij} for a selection of trial radii, and finding the R which corresponds to the eigenvalue 1. In practice this is the largest eigenvalue. In supplementary Fig. 1 we do this for a variety of grid sizes N_{grid}. As can be seen, the value of $R = 1.16$ produces an eigenvalue closest to 1. This is in reasoable agreement with $R_{sim} \approx 1.15$ as measured from simulation data (for simulation parameters, see the caption of supplementary Fig. 1). Also shown in supplementary Fig. 1 is the eigenvector g_i which approximates the radial density $g(r)$.

Determining R and $g(r)$ this way satisfies supplementary equation (7). Equation supplementary equation (6) must also be satisfied, which we checked by substitution.

Radii with unit vector attraction kernel. While we were unable to calculate the density of the static sync and async states analytically, we were able to partially calculate the radii of its support using dimensional analysis. We assume that the radii are determined when the magnitudes of attractive and repulsive forces balance. For the sync state

$$\mathbf{I}_{\rm att}(R_{\rm sync}) F(\theta) \propto \mathbf{I}_{\rm rep}(R), \tag{10}$$
$$(1)(1+J) \propto 1/R_{\rm sync},$$
$$R_{\rm sync} = C(1+J)^{-1},$$

where C is an unknown constant. Note the effective spatial attraction force is $\mathbf{I}_{\text{att}}(\mathbf{x})F(\theta)$, and $F(\theta) = 1 + J\cos(0) = 1 + J$ in the static sync state (since all swarmalators have the same phase). In the static async state the calculation is the same with $F(\theta) = 1$ giving

$$R_{\text{async}} = C. \tag{11}$$

We cannot find the radii directly because of the unknown constant C. Their ratio is however given by

$$\frac{R_{\text{sync}}}{R_{\text{async}}} = \frac{1}{1+J} \tag{12}$$

which agrees with simulation as shown in supplementary Fig. 2.

SUPPLEMENTARY NOTE 2

Analytic derivation. We calculate the stability of the static async state by considering a linear perturbation η in density space,

$$\rho(\underline{x}, t) = \rho_0(\underline{x}) + \epsilon\eta(\underline{x}, t), \tag{13}$$

where

$$\rho_0(\underline{x}) = (4\pi^2)^{-1} g(r), \qquad |\mathbf{x}| < R \tag{14}$$

is the unperturbed density and $\underline{x} := (\mathbf{x}, \theta)$. The decomposition given by supplementary equation (13) of the density induces a decomposition in $\underline{v}$,

$$\underline{v} = \underline{v}^{(0)} + \epsilon\underline{v}^{(1)} = \epsilon\underline{v}^{(1)}, \tag{15}$$

where $\underline{v}^{(0)} \equiv \underline{0}$ since swarmalators are motionless in the static async state. By normalizing supplementary equation (13) we require

$$\int \eta(\underline{x}, t) d\underline{x} = 0. \tag{16}$$

We substitute the ansatz given by supplementary equation (13) into the continuity equation for $\rho(\underline{x}, t)$ to derive an evolution equation for η. Collecting terms at $O(\epsilon)$ gives

$$\dot{\eta} + \rho_0 \nabla.\underline{v}^{(1)} + \underline{v}^{(1)}.\underline{\nabla}\rho_0 = 0. \tag{17}$$

We first calculate the divergence $\nabla.\underline{v}^{(1)} = \partial_{\mathbf{x}}\mathbf{v}_{\mathbf{x}} + \partial_\theta v_\theta$. Recalling that

$$\nabla_{\mathbf{x}}.\frac{\tilde{\mathbf{x}} - \mathbf{x}}{|\tilde{\mathbf{x}} - \mathbf{x}|} = -\frac{1}{|\tilde{\mathbf{x}} - \mathbf{x}|}, \tag{18}$$

we see the terms on the LHS are given by

$$\partial_{\mathbf{x}}\mathbf{v}_{\mathbf{x}}(\mathbf{x}, \theta, t) = -\int \left(\frac{1}{|\tilde{\mathbf{x}} - \mathbf{x}|}\Big(1 + J\cos(\tilde{\theta} - \theta)\Big) - 2\pi\delta(\tilde{\mathbf{x}} - \mathbf{x}) \right) \eta(\tilde{\mathbf{x}},\ \tilde{\theta}, t)\ d\tilde{\mathbf{x}}\, d\tilde{\theta} \tag{19}$$

$$\partial_\theta v_\theta(\mathbf{x}, \theta, t) = -K \int \frac{\cos(\tilde{\theta} - \theta)}{|\tilde{\mathbf{x}} - \mathbf{x}|} \eta(\tilde{\mathbf{x}}, \tilde{\theta}, t) d\tilde{\mathbf{x}}\, d\tilde{\theta}. \tag{20}$$

We simplify these by expanding $\eta(\mathbf{x}, t)$ in a Fourier series over θ,

$$\eta(\mathbf{x}, \theta, t) = \sum_{n=0}^{\infty} b_n(\mathbf{x}, t) e^{in\theta} + c.c., \tag{21}$$

where 'c.c.' denotes the complex conjugate. Plugging this ansatz into supplementary equations (19) and (20) and performing the integration over θ leads to

$$\underline{\nabla}.\underline{v}^{(1)} = \Big(-2\pi b_0(\mathbf{x},t) + \int \frac{b_0(\tilde{\mathbf{x}},t)}{|\tilde{\mathbf{x}}-\mathbf{x}|}\, d\tilde{\mathbf{x}} \Big) - e^{i\theta}\Big(\frac{J+K}{2} \int \frac{b_1(\tilde{\mathbf{x}},t)}{|\tilde{\mathbf{x}}-\mathbf{x}|}\, d\tilde{\mathbf{x}} \Big) \tag{22}$$

Note the appearance of the $1/|\tilde{\mathbf{x}}-\mathbf{x}|$ in the first term of supplementary equation (19) and in (20). This tells us that the divergence of the spatial attraction and phase attraction have the same length scale. This is a convenient property, which results from our careful choosing of the unit vector attraction $\mathbf{I}_{\text{att}}(\mathbf{x}) = \mathbf{x}/|\mathbf{x}|$, so that $\nabla.\mathbf{I}_{\text{att}}(\mathbf{x}) = H(\mathbf{x}) = 1/|\mathbf{x}|$.

We next calculate the third term, $\underline{v}^{(1)}.\underline{\nabla\rho_0}$, in the ODE for η given by supplementary equation (17). Note that since $\rho_0 = (4\pi)^{-1}g(r)$ for $r < R$, then the gradient is purely in the spatial direction $\underline{\nabla\rho_0} = \nabla\rho_0 + \partial_\theta\rho_0 = \nabla\rho_0$, which we leave in cartesian coordinates. Thus we only need to calculate the spatial components of $\underline{v}^{(1)} = \mathbf{v}_{\mathbf{x}}^{(1)} + v_\theta$. Plugging the Fourier ansatz into the expression for $\mathbf{v}_{\mathbf{x}}^{(1)}$ and simplifying gives

$$\mathbf{v}_{\mathbf{x}}^{(1)} = \int \Big(\frac{\tilde{\mathbf{x}}-\mathbf{x}}{|\tilde{\mathbf{x}}-\mathbf{x}|} - \frac{\tilde{\mathbf{x}}-\mathbf{x}}{|\tilde{\mathbf{x}}-\mathbf{x}|^2} \Big) b_0(\tilde{\mathbf{x}},t)\, d\tilde{\mathbf{x}} + e^{i\theta}\frac{J}{2}\int \frac{\tilde{\mathbf{x}}-\mathbf{x}}{|\tilde{\mathbf{x}}-\mathbf{x}|} b_1(\tilde{\mathbf{x}},t)\, d\tilde{\mathbf{x}}. \tag{23}$$

Putting our results supplementary equations (22) and (23) into the ODE given by supplementary equation (17) for η and collecting terms at each harmonic $e^{in\theta}$ leads to

$$\dot{b}_0(\mathbf{x},t) = -\nabla\rho_0(\mathbf{x}).\int \Big(\frac{\tilde{\mathbf{x}}-\mathbf{x}}{|\tilde{\mathbf{x}}-\mathbf{x}|} - \frac{\tilde{\mathbf{x}}-\mathbf{x}}{|\tilde{\mathbf{x}}-\mathbf{x}|^2} \Big) b_0(\tilde{\mathbf{x}},t)\, d\tilde{\mathbf{x}} - \rho_0(\mathbf{x})\Big(2\pi b_0(\mathbf{x},t) - \int \frac{b_0(\tilde{\mathbf{x}},t)}{|\tilde{\mathbf{x}}-\mathbf{x}|} d\tilde{\mathbf{x}} \Big) \tag{24}$$

$$\dot{b}_1(\mathbf{x},t) = -\frac{J}{2}\,\nabla\rho_0(\mathbf{x}).\int \frac{\tilde{\mathbf{x}}-\mathbf{x}}{|\tilde{\mathbf{x}}-\mathbf{x}|} b_1(\tilde{\mathbf{x}},t)\, d\tilde{\mathbf{x}} + \frac{(J+K)}{2}\rho_0(\mathbf{x})\int \frac{1}{|\tilde{\mathbf{x}}-\mathbf{x}|} b_1(\tilde{\mathbf{x}},t) d\tilde{\mathbf{x}}, \tag{25}$$

$$\dot{b}_n(\mathbf{x},t) = 0, \qquad n > 1. \tag{26}$$

We see the $n = 0, 1$ modes are distinguished. Notice the parameters J, K appear only in the equation for $b_1(\mathbf{x},t)$. This tells us that this is the mode which determines the stability of the static async state, since the state becomes unstable as these parameters are varied. We thus study $b_1(\mathbf{x},t)$, by expanding in a Fourier series: $b_1(r,\phi,t) = \sum_{m=0}^{\infty} f_m(r,t)e^{im\phi} + c.c.$. Substituting this ansatz into supplementary equation (25) and doing some algebra leads to an evolution equation for each mode $f_m(r,t)$. We then set $f_m(r,t) = e^{\lambda_m t}c_m(r)$ which leads to the following eigenvalue equation for each mode:

$$\lambda_m c_m(r) = \int_0^R H_m(r,s)c_m(s)s\, ds, \tag{27}$$

where the R is the radius of the support of the density in the static async state. This is the equation that appears in the main text. There, we also plot the real parts of the eigenvalues $\lambda_m^*{}'(K)$ for the five most unstable modes, for $J = 0.5$. For convenience we replot this in supplementary Fig. 3.

Finite-size scaling of eigenvalues. As can be seen in supplementary Fig. 3, $\lambda_m^*{}'(K)$ are small but positive for $K < K_c$. But are these positive values genuine? They could be artifacts of the discretization scheme used to approximate $\lambda_m^*(K)$, which has a parameter N' representing the number of grid points used to approximate the integral in supplementary equation (27). There is no reason to believe that $\lambda_m^*{}'$ remain positive as $N' \to \infty$; they could become zero, or negative, giving rise to different stability properties.

Hence we perform a finite-size scaling analysis of $\lambda_m^*{}'(K)$. In supplementary Fig. 4 we show $\lambda_0^*{}'(K)$ for $K < K_c$ for different N'. As N' is increased, $\lambda_0^*{}'$ gets progressively smaller while remaining positive, supporting the claim that the modes f_m are unstable. We next used two crude approaches to probe the $N' \to \infty$ limit. First, we fit the data to a function of the form $a + bh^c$, where $h = 1/N'$. In supplementary Fig. 5 we show the results of this procedure for $K = -0.5$ and $K = -0.8$. As shown $\lambda_0^*(h \to 0)$ attains small but *negative* values. Second, we used Richardson extrapolation, a method used to estimate the limiting value of a converging sequence. Using the values of $\lambda_0^*{}'$ at $h = 1/400, 1/800, 1/1600$ it gave the approximation $\lambda_0^* = +2 \times 10^{-8}$ for $K = -0.5$ and $\lambda_0^* = -4 \times 10^{-5}$ for $K = -0.8$.

Confusingly, the two methods give approximations with different signs for $K = -0.5$. Furthermore, the magnitudes of the estimates are very small. These two facts make the results rather unconvincing. We thus declare

the finite-size scaling analysis inconclusive; we have not been able to satisfactorily determine the sign of ${\lambda_m^*}'$ as $N' \to \infty$. This prevents us from determining the stability properties of the static async state. The only thing we can say with confidence is that there is a parameter regime $K < K_c$ where the state has *weak* stability properties, since here, their signs notwithstanding, the magnitudes of the eigenvalues are very small. For $K > K_c$, however, the eigenvalues become unambiguously positive, explicitly indicating the instability of static async state in this regime.

Critical coupling K_c. In the main text we approximated the critical coupling separating these two regimes as $K_c = \min_m K_m^* = K_2^*$, where K_m^* marks the point where ${\lambda_m^*}'$ starts to increase significantly. For example $K_1^* \approx -0.6$ as seen in supplementary Fig. 3. We use the following definition:

$$K_m^* = \operatorname{argmin} \frac{d^2 {\lambda_m^*}'}{dK^2}. \tag{28}$$

We calculated K_c using supplementary equation (28) for various values of J and plot the results in supplementary Fig. 6. The points fall on the straight line

$$K_c = -1.2\ J. \tag{29}$$

To test this prediction we calculated K_c from simulation data by finding the point at which the order parameter $S = \max(S_+, S_-)$ bifurcates from zero. We define this K_c to be the midpoint of the interval (K_i, K_{i+1}) over which the derivative of S with respect to K is largest. We did this over a range of J, the results of which are the black dots in the phase diagram in the main text Fig. 1.

Finite-size scaling of S. Measuring K_c this way depends on the population size N. To get a feel for the scale of this dependence, and to see if any scaling laws are present, we perform a finite-size scaling analysis of S and K_c. Given the computational cost of each simulation, and the necessity of collecting many realizations at each parameter value, the quality of the data we collected was limited. Hence, our analysis is preliminary.

In supplementary Fig. 7 we show $S(K)$ for different N. The qualitative shape of the curves do not change, but the bifurcation point K_c increases with N. In supplementary Fig. 8 we show $K_c(N)$. No trend is evident. We tried to fit the data to a curve of the form $a + bh^c$, but the fitting algorithms failed. This is not too surprising given the sparsity and quality of the data. Next we tried $a + bh^{1/2}$, which gave the black curve shown in the Figure. The parameter of best fit a, representing $K_c = N \to \infty$, has confidence interval $(-0.47, -0.59)$.

SUPPLEMENTARY NOTE 3

As discussed in the main text, when white noise is added to the phase dynamics, the static async state is no longer static in the sense that the swarmalators are no longer motionless. They oscillate in space and phase, and thus we call the new state the noisy async state. In the continuum limit, the spatial movements should vanish. In supplementary Fig. 9 we demonstrate this by plotting the time-averaged, mean population spatial velocity, $\langle v_x \rangle = \langle \frac{1}{N} \sum_j \sqrt{\dot{x}_j^2 + \dot{y}_j^2} \rangle_t$ where $\langle . \rangle_t$ is the time average, as a function of K for $J = 0.5$ and $D_\theta = 0.06$. As can be seen, there is a sharp drop in the velocity at a critical value of K indicating the transition to the active phase wave state. In this state, the spatial velocity decays to zero with increasing population size like $N^{-1/2}$, as indicated by the downward arrow. We show $\langle v_x \rangle$ versus N for a fixed value of K in supplementary Fig. 10, in which the $N^{-1/2}$ dependence is clear. We have fitted the data to a curve of form $a + bN^{-c}$, plotted along with the data.

SUPPLEMENTARY NOTE 4

The model we introduced in this work is

$$\dot{\mathbf{x}}_{\mathbf{i}} = \frac{1}{N} \sum_{j \neq i}^{N} \mathbf{I}_{\text{att}}(\mathbf{x_j} - \mathbf{x_i}) F(\theta_j - \theta_i) - \mathbf{I}_{\text{rep}}(\mathbf{x_j} - \mathbf{x_i}), \tag{30}$$

$$\dot{\theta}_i = \omega_i + \frac{K}{N} \sum_{j \neq i}^{N} H(\theta_j - \theta_i) G(\mathbf{x_j} - \mathbf{x_i}). \tag{31}$$

We studied the following instance of this model:

$$\dot{\mathbf{x_i}} = \frac{1}{N}\sum_{j\neq i}^{N} \frac{\mathbf{x_j}-\mathbf{x_i}}{|\mathbf{x_j}-\mathbf{x_i}|}\Big(1+J\cos(\theta_j-\theta_i)\Big) - \frac{\mathbf{x_j}-\mathbf{x_i}}{|\mathbf{x_j}-\mathbf{x_i}|^2}, \tag{32}$$

$$\dot{\theta}_i = \frac{K}{N}\sum_{j\neq i}^{N} \frac{\sin(\theta_j-\theta_i)}{|\mathbf{x_j}-\mathbf{x_i}|}. \tag{33}$$

To check if the phenomena we found are generic for the model, as opposed to being specific to the *instance* of the model, we ran simulations for the different choices of the functions $\mathbf{I}_{\text{att}}(\mathbf{x}), \mathbf{I}_{\text{rep}}(\mathbf{x}), G(\mathbf{x})$ defined by supplementary equations (34)-(38):

$$\mathbf{I}_{\text{att}}(\mathbf{x}),\ \mathbf{I}_{\text{rep}}(\mathbf{x}),\ G(\mathbf{x}) = \frac{\mathbf{x}}{|\mathbf{x}|^2},\ \frac{\mathbf{x}}{|\mathbf{x}|^4},\ \frac{1}{|\mathbf{x}|^3} \tag{34}$$

$$= \frac{\mathbf{x}}{|\mathbf{x}|^2},\ \frac{\mathbf{x}}{|\mathbf{x}|^4},\ \frac{1}{|\mathbf{x}|} \tag{35}$$

$$= \frac{\mathbf{x}}{|\mathbf{x}|^2},\ \frac{\mathbf{x}}{|\mathbf{x}|^4},\ \exp(-|\mathbf{x}|^2/\nu) \tag{36}$$

$$= \mathbf{x}\exp(-|\mathbf{x}|^2/\nu),\ \frac{\mathbf{x}}{|\mathbf{x}|^2},\ \frac{1}{|\mathbf{x}|} \tag{37}$$

$$= \mathbf{x},\ \frac{\mathbf{x}}{|\mathbf{x}|^2},\ \frac{1}{|\mathbf{x}|} \tag{38}$$

In all these cases, we found the same phenomena, as evidenced by the behavior of the order parameters for these new instances of the model, shown in supplementary Fig. 11.

The first two changes were to check if our phenomena were dependent on length scale. In the modification given by supplementary equation (34) we replaced the physically unrealistic unit vector attraction kernel with one that spatially decays. We chose a power law with exponent -1. To keep the length scale of $\mathbf{I}_{\text{rep}}(\mathbf{x})$ shorter than that of $I_{\text{att}}(\mathbf{x})$, we changed the latter's exponent to -3. Further, to keep the length scale of the phase dynamics (captured by the function $G(\mathbf{x})$) the same as that of $\mathbf{I}_{\text{rep}}(\mathbf{x})$, as it is in the original model, we changed the exponent of $G(\mathbf{x})$ to -3. In the modification given by supplementary equation (35), we matched the exponents of $\mathbf{I}_{\text{att}}(\mathbf{x})$ and $G(\mathbf{x})$, so that the length scale of the phase dynamics was the same as that of spatial attraction. As displayed in panels (a) and (b) of supplementary Fig. 11, these changes led to no new behavior.

The purpose of the next two modifications, given by supplementary equations (36) and (37), was to check if the phenomena were independent of specific functional forms. To this end, we replaced the power-law choices for $\mathbf{I}_{\text{att}}(\mathbf{x})$ and $G(\mathbf{x})$ with exponentials with tunable length scales ν. For the modification given by supplementary equation (36) we varied ν from 0 to 1 and found the same phenomena. For the modification given by supplementary equation (37), we found that for $\nu \gtrsim 1$, the same phenomena were realized. However when $\nu \lesssim 1$, only the static sync and static async states were found. The theoretical reasons for this are not yet understood, and are left for future work.

Non-stationary phase waves. The final modification given by (38) corresponds to the original model with the unit vector attraction kernel replaced by a linear kernel. While a linear function is somewhat unrealistic, physically speaking, it has the advantage of being much easier to analyze. In particular, in combination with the newtonian repulsion, it leads to swarmalators being confined to disks of uniform density in the static sync and static async states. As we showed in the Methods section in the main text, this let us solve for the radii of the support disks in these states, as well as letting us solve for the density of the static phase wave state, along with its inner and outer radii.

The disadvantage of the linear attraction kernel is that it has non-generic behavior, which we why we chose not to present it in the main text. Its non-genericity is such that it leads to extra states not found in other instances of the swarmalator model. We call all these new states 'non-stationary phase waves', since (in contrast to states we have studied so far) they correspond to non-stationary densities of the swarmalators. As K is decreased, the non-stationary phase waves bifurcate from the active phase wave, before ultimately morphing into the static async state, as shown in a plot of the order parameters in supplementary Fig. 14. During this transition, the phase $\Psi_\pm$ of the order parameter $W_\pm = S_\pm e^{i\Psi_\pm} = N^{-1}\sum_j e^{i(\phi_j \pm \theta_j)}$ changes from being constant to being time-dependent. For values of K near the active phase wave, $W_\pm$ rotates uniformly, so that $\Psi = \Omega t$, as shown in supplementary Fig. 12(b). At the swarmalator

level, this means the correlation between the spatial angle ϕ and phase θ becomes periodic, namely $\theta \approx \pm\phi + C(t)$, as illustrated by the moving bands in (ϕ, θ) space shown in supplementary Fig. 13 and in Supplementary Movie 8.

For more negative K, wilder versions of the traveling phase wave are encountered. Here, both $S_{\pm}$ and $\Psi_{\pm}$ start to oscillate irregularly, as shown in supplementary Fig. 12(c). The precise nature of this irregularity is not yet understood theoretically, and is left for future work. As K is further decreased, the average amplitude of S smoothly decreases to zero, at which point the static async state is achieved, as shown in supplementary Fig. 12(d).

To distinguish between the active, and non-stationary, phase wave states, we define the order parameter Γ. This is 1 if $\Psi_{\pm}$ has executed at least one cycle, after transients have been discarded. In conjunction with the order parameters $S_{\pm}$ and γ, this lets us discern all the states as K is varied, as shown in supplementary Fig. 14.

SUPPLEMENTARY NOTE 5

In the main text we explored the behavior of our model in three dimensions for identical swarmalators with no noise. We plotted four of the five states in the (x, y, z) plane, where each swarmalator was colored according to its phase. Here we show these same four states as scatter plots in (ϕ, θ) plane, where ϕ is the azimuthal angle of swarmalators $\phi = \tan^{-1}(y/x)$. supplementary Fig. 15 shows the results, where the points have been colored according to their polar angle $\alpha = \cos^{-1}(\frac{z}{\sqrt{x^2+y^2+z^2}})$. The plots look qualitatively the same as those obtained for the 2D model. We also show the states in the (θ, α) plane, and color particles according to their polar angle, in supplementary Fig. 16.

The 3D analogues of the order parameters S and γ also have the same qualitative behavior as their 2D versions, as seen in supplementary Fig. 17.

The same is true of the radii of the spherical densities of the static async and static async states, which in the main text we showed were $R_{\text{async}} = 1$ and $R_{\text{sync}} = (1 + J)^{-1/3}$. Prohibitively large population sizes would be needed to confirm these predictions in detail. To get around this, we instead investigated the finite-size scaling behavior of the radii. In supplementary Fig. 18(a) we show R_{async} for progressively larger population sizes N. As can be seen, the data appear to converge to the theoretical value of 1. We confirmed this convergence by fitting the data to a curve of the form $a + bN^{-c}$ using Mathematica, which returned a best fit parameter of $a = 0.998$. In supplementary Fig. 18(b), we plot R_{sync} versus J. As before, for each each value of J, the radius $R_{\text{sync}}(N)$ was calculated for increasing population sizes N, from which the $N \to \infty$ limit was found by fitting the data to a curve $a + bN^{-0.5}$, and identifying $a = R_{\text{sync}}$. As can be seen, simulation and theory agree

We next checked robustness to disorder. As in 2D, all but the splintered phase wave state persist in the presence of noise and distributed natural frequencies. We show these states in supplementary Fig. 19

SUPPLEMENTARY NOTE 6

In our work so far, swarmalators have been confined to the (x, y) plane. Our motivation for this was physical realism. However, as we discovered, the steady states of our system were often one dimensional, displaying circular symmetry. This raises the question: could we recover the same phenomena by confining swarmalators to move on a circle? In this section, we show that we can.

The 1D governing equations are

$$\dot{x_i} = \frac{1}{N}\sum_{j=1}^{N} I_{\text{att}}(x_j - x_i)F(\theta_j - \theta_i) - I_{\text{rep}}(x_j - x_i), \tag{39}$$

$$\dot{\theta}_i = \omega_i + \frac{K}{N}\sum_{j=1}^{N} H_{\text{att}}(\theta_j - \theta_i)G(x_j - x_i), \tag{40}$$

where x_i is an angle parameterizing the position of swarmalator i on the unit circle, and the various functions are as

described in the main text. We consider the following instance of this model:

$$\dot{x_i} = \frac{A}{N}\sum_{j=1}^{N}\sin(x_j - x_i)\Big[B + J\cos(\theta_j - \theta_i) - C\Big(\frac{1+\cos(x_j - x_i)}{2}\Big)^n\Big] \tag{41}$$

$$\dot{\theta}_i = \omega + \frac{K}{N}\sum_{j=1}^{N}\sin(\theta_j - \theta_i)\Big(\frac{1+\cos(x_j - x_i)}{2}\Big)^n. \tag{42}$$

The parameters J, K are the same as those in the main text. We choose the sine function for H_{att} and I_{att} for simplicity, again in the spirit of the Kuramoto model. The function $H(x) = 2^{-n}(1+\cos x)^n$ represents a smooth pulse of unit strength. It contains the parameter n, a positive integer that controls the width of the pulse. Increasing n decreases the width, as shown in supplementary Fig. 20. The function $F(\theta)$ is also shown.

The remaining parameters are A, B, C and the natural frequency ω. By rescaling time and going to a rotating frame we set $A = 1$ and $\omega = 0$ W.L.O.G.. To keep our model minimal, we wish to fix B, C, which control the strength of the attractive and repulsive spatial forces. We want I_{rep} to mimic hard-shell repulsion. To this end, we need $C > B$. By experimenting numerically, we find $B = 1$ and $C = 10$ produced the desired behavior. For smaller values of C, swarmalators collapse to a single point x. Lastly, our simulations also indicate that the phenomena do not depend on the spatial length scale n. We therefore also fix $n = 1$, unless otherwise stated.

Phenomena. To investigate the behavior of our system, we again performed numerical experiments using python's ODE solver 'odeint'. We selected the initial positions and phases of the swarmalators uniformly at random from $[-\pi, \pi]$. We find the system settles into 1D versions of the five states found in 2D. supplementary Figure 21 shows the phase diagram, which is qualitatively the same as the Fig. 1 in the main text. The 1D stationary states are shown in supplementary Fig. 22 and panels (a) and (b) of supplementary Fig. 23, and are direct analogues of their 2D versions.

The non-stationary states are shown in supplementary Fig. 24 and panels (c) and (d) of supplementary Fig. 23. These are different from their 2D analogues, in that the density $\rho(x, \theta, t)$ is now non-stationary: in the splintered phase wave state, the clusters of similarly-phased swarmalators gently expand and contract in time. These contractions are also present in the active phase wave state, but are now more violent, as indicated in supplementary Fig. 23(d).

Analysis. As before, the order parameters $S_\pm$ and γ together let us discriminate between all the states of our systems, as illustrated in supplementary Fig. 25. At $K = K_1(J)$, we see a sharp transition in γ, identifying the change from the splintered phase wave to the active phase wave. At $K = K_2(J)$, we see both γ and $S_\pm$ drop to zero, signaling the transition to the static async state. After the following subsection, we again perform a linear stability analysis in density space to find K_2 analytically,

$$K_2(n, J) = -\frac{\sqrt{\pi}}{2}\frac{\Gamma(n)}{\Gamma(n+1/2)}(n+1)\,J \tag{43}$$

which for $n = 1$ reduces to the line $K_2 = -2J$ drawn in supplementary Fig. 21.

Genericity. To check if our phenomena are generic, we ran simulations for different choices of the functions $I_{\text{rep}}(x), F(\theta), G(x)$, summarized below:

$$I_{\text{rep}}(x) = \frac{1}{C - \cos(x)}, \tag{44}$$

$$F(\theta) = \exp(-\frac{\sin(\theta/2)^2}{2\nu}), \tag{45}$$

$$G(x) = \exp(-\frac{\sin(x/2)^2}{2\nu}). \tag{46}$$

In all cases, the phenomena we found were the same. In contrast to the 2D swarmalators, we could not find a 1D realization of the traveling phase wave states. supplementary Figure 26 below shows the order parameters S, γ for the $G(x)$ given by supplementary equation (46). As can be seen, their behavior is qualitatively the same as that in supplementary Fig. 25 above.

Stability of static async state in 1D. We investigate the stability of the static async state by performing a linear stability analysis in density space. The analysis is the same as for the 2D case. Let

$$\rho(x,\theta,t) = \rho_0(x,\theta,t) + \epsilon\eta(x,\theta,t) \tag{47}$$

as before, except now $\rho_0 = (4\pi^2)^{-1}$. Substituting this ansatz into the continuity equation and collecting terms at $O(\epsilon)$ gives

$$\dot{\eta} + \rho_0 \underline{\nabla}.\underline{v}^{(1)} = 0. \tag{48}$$

We expand $\eta(x,\theta,t)$ in a Fourier series,

$$\begin{aligned} \eta(x,\theta,t) = \sum_{m=0,l=0} & \alpha_{m,l}(t)\cos(mx)\cos(l\theta) \\ & + \beta_{m,l}(t)\sin(mx)\sin(l\theta) \\ & + \gamma_{m,l}(t)\cos(mx)\sin(l\theta) \\ & + \delta_{m,l}(t)\sin(mx)\cos(l\theta). \end{aligned} \tag{49}$$

Note the zero modes $\alpha_{0,0}, \beta_{0,0}\dots$ are zero via the normalization condition $\int \eta(x,\theta,t)dx\,d\theta = 0$ (but the $\alpha_{0,1}, \alpha_{1,0}\dots$ aren't necessarily). Computing the divergence, and plugging the result into supplementary equation (48), and projecting onto the various basis vectors $\cos(mx)\cos(l\theta),\dots$ leads to the ODEs for the Fourier components $\alpha_{m,l}, \beta_{m,l}, \gamma_{m,l}, \delta_{m,l}$. The calculation is difficult to achieve for a general n, so we instead tabulate the results for $n = 1, 2, \dots$). The results for $n = 1$ are

$$\begin{aligned} &\dot{\alpha}_{0,1} + \tfrac{1}{2}(B-2)\alpha_{0,1} = 0 && \dot{\delta}_{1,0} - \tfrac{1}{2}K\delta_{1,0} = 0 \\ &\dot{\gamma}_{0,1} + \tfrac{1}{2}(B-2)\gamma_{0,1} = 0 && 8\dot{\alpha}_{1,1} - \alpha_{1,1}(2J+K) = 0 \\ &\dot{\alpha}_{0,2} + \tfrac{1}{2}B\alpha_{0,2} = 0 && 8\dot{\beta}_{1,1} - \beta_{1,1}(2J+K) = 0 \\ &\dot{\gamma}_{0,2} + \tfrac{1}{2}B\gamma_{0,2} = 0 && 8\dot{\gamma}_{1,1} - \gamma_{1,1}(2J+K) = 0 \\ &\dot{\alpha}_{1,0} - \tfrac{1}{2}K\alpha_{1,0} = 0 && 8\dot{\delta}_{1,1} - \delta_{1,1}(2J+K) = 0 \end{aligned} \tag{50}$$

where we have omitted all equations of the form $\dot{\alpha}_{l,m} = 0$, $\dot{\beta}_{l,m} = 0$, etc . Looking at the $\alpha_{0,1}, \gamma_{0,1}$ modes, we see the static async is stable when $B > 2$

We remind the reader that B is the strength of the spatial repulsion. So this condition is telling us that B needs to be sufficiently large for the static async state to exist. From the $\alpha_{1,0}, \delta_{1,0}$ and $\alpha_{0,2}, \gamma_{0,2}$ modes, we see we also require $K < 0$, and $B > 0$ respectively for the static async to be stable. These conditions are trivially satisfied in the parameter regime of interest.

Looking at the $\alpha_{1,1}, \beta_{1,1},\dots$ modes finally gives us the desired critical coupling strength:

$$K_2(n=1) = -2J. \tag{51}$$

We next repeated the calculation for $n = 2, 3\dots$, and then using Mathematica's "FindSequenceFunction", found the critical coupling K_2 for a general n:

$$K_2(n) = -\frac{\sqrt{\pi}}{2}\frac{\Gamma(n)}{\Gamma(n+1/2)}(n+1)\,J. \tag{52}$$
