# Supplementary material for: Oscillators that sync and swarm
Source: Nat Commun. 2017 Nov 15;8:1504. doi: 10.1038/s41467-017-01190-3 (PMC5686229; doi:10.1038/s41467-017-01190-3)
Supplement: Supplementary file 3 — Description of Additional Supplementary Files [file 41467_2017_1190_MOESM3_ESM.pdf]

Description of Additional Supplementary Files

File Name: Supplementary Movie 1
Description: Evolution to static sync state for (J, K) = (0.1, 1)

File Name: Supplementary Movie 2
Description: Evolution to static async state for (J, K) = (0.1, -1)

File Name: Supplementary Movie 3
Description: Evolution to static phase wave state for (J, K) = (0.1, -1)

File Name: Supplementary Movie 4
Description: Evolution to splintered phase wave state for (J, K) = (1.0, -0.1)

File Name: Supplementary Movie 5
Description: Evolution to active phase wave state for (J, K) = (1.0, -0.75)

File Name: Supplementary Movie 6
Description: Evolution to splintered phase wave state in the (phi, theta) plane for (J, K) = (1.0, -0.1)

File Name: Supplementary Movie 7
Description: Evolution to active phase wave state in the (phi, theta) plane (J, K) = (1.0, -0.75)

File Name: Supplementary Movie 8
Description: Evolution to non-stationary phase wave state in the (phi, theta) plane (J, K) = (1.0, -0.75)

File Name: Supplementary Movie 9
Description: Evolution to the static async state in 3D for (J, K) = (0.5, -1)

File Name: Supplementary Movie 10
Description: Evolution to the static phase wave state in 3D for (J, K) = (0.5, 0)

File Name: Supplementary Movie 11
Description: Evolution to the splintered phase wave state in 3D for (J, K) = (0.5, -0.05)

File Name: Supplementary Movie 12
Description: Evolution to the active phase wave state in 3D for (J, K) = (0.5, -0.6)
