# Supplementary figures and images for: Oscillators that sync and swarm
Source: Nat Commun. 2017 Nov 15;8:1504. doi: 10.1038/s41467-017-01190-3 (PMC5686229; doi:10.1038/s41467-017-01190-3)

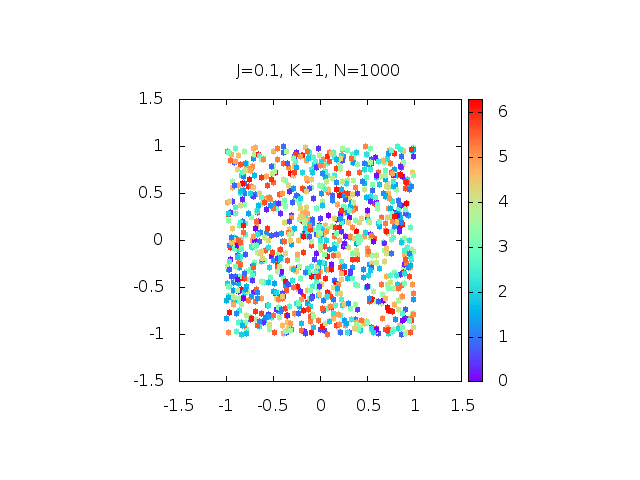

Supplement: Supplementary file 4 — Supplementary Movie 1 [file 41467_2017_1190_MOESM4_ESM.gif]

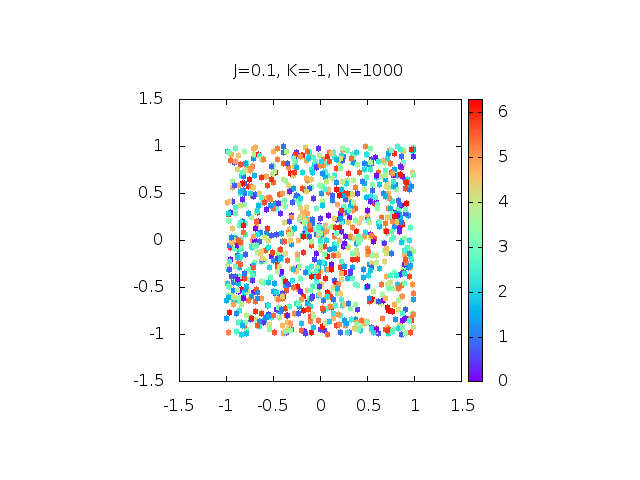

Supplement: Supplementary file 5 — Supplementary Movie 2 [file 41467_2017_1190_MOESM5_ESM.gif]

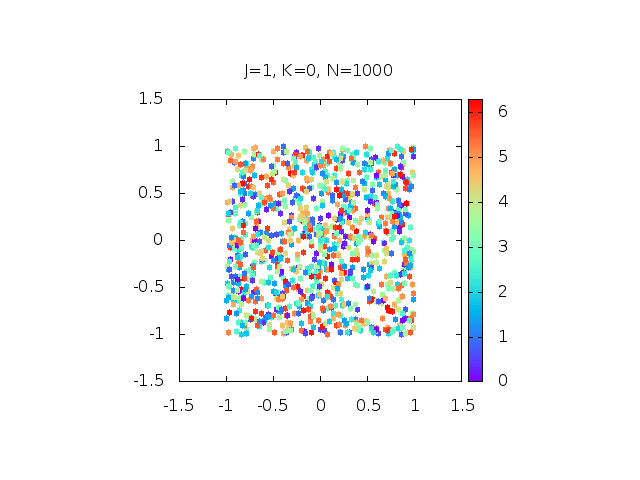

Supplement: Supplementary file 6 — Supplementary Movie 3 [file 41467_2017_1190_MOESM6_ESM.gif]

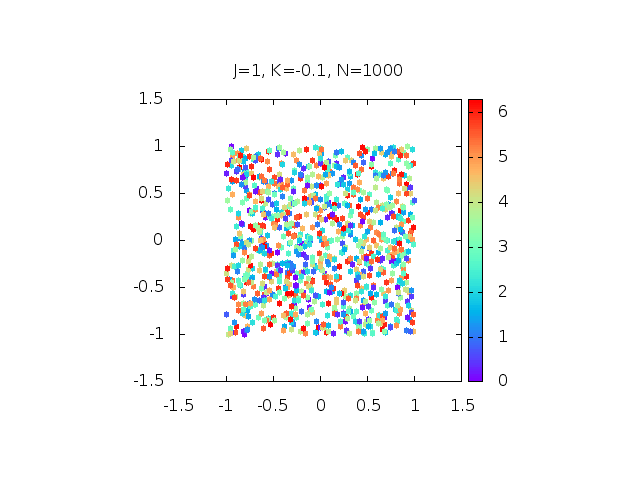

Supplement: Supplementary file 7 — Supplementary Movie 4 [file 41467_2017_1190_MOESM7_ESM.gif]

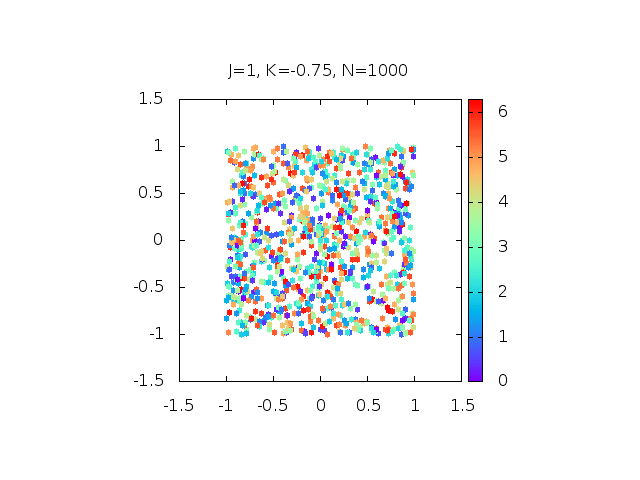

Supplement: Supplementary file 8 — Supplementary Movie 5 [file 41467_2017_1190_MOESM8_ESM.gif]

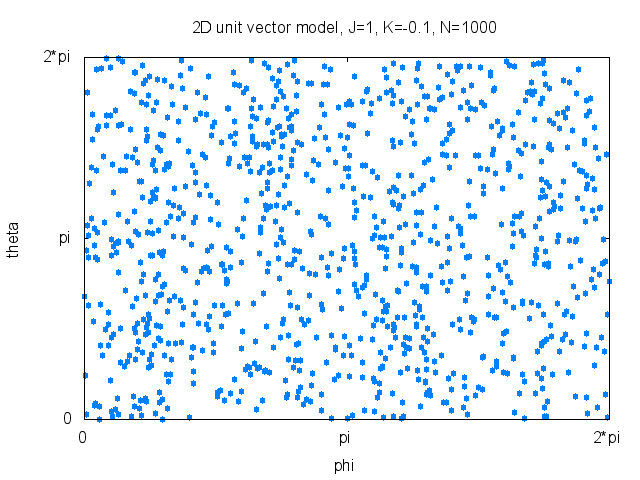

Supplement: Supplementary file 9 — Supplementary Movie 6 [file 41467_2017_1190_MOESM9_ESM.gif]

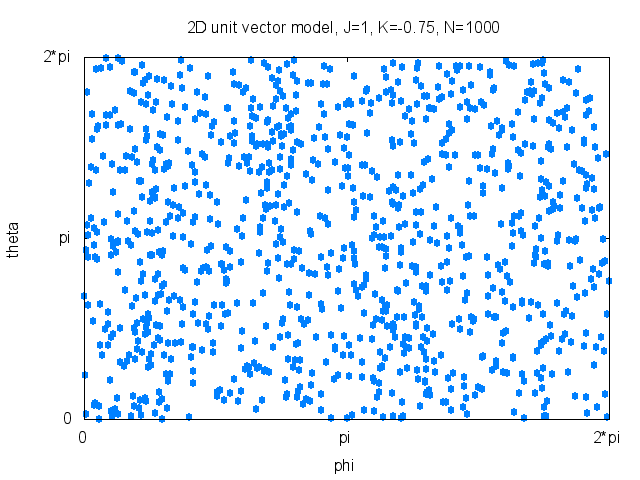

Supplement: Supplementary file 10 — Supplementary Movie 7 [file 41467_2017_1190_MOESM10_ESM.gif]

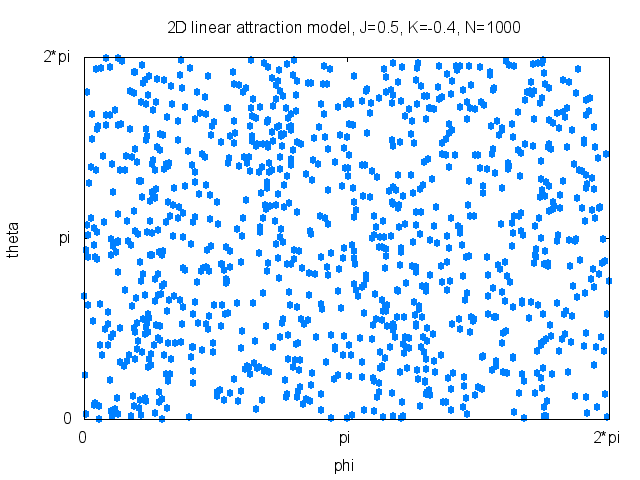

Supplement: Supplementary file 11 — Supplementary Movie 8 [file 41467_2017_1190_MOESM11_ESM.gif]

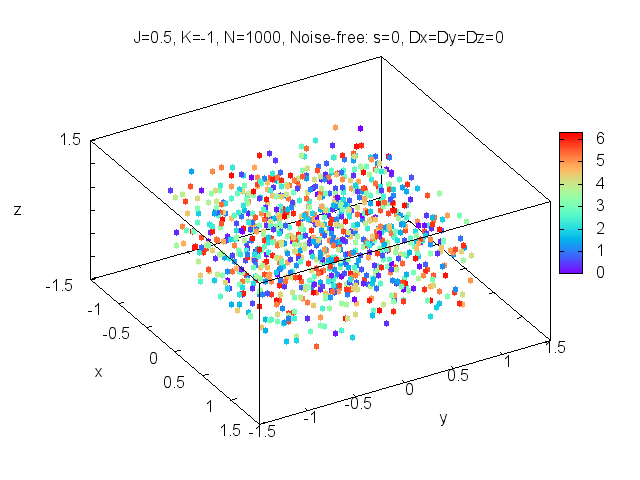

Supplement: Supplementary file 12 — Supplementary Movie 9 [file 41467_2017_1190_MOESM12_ESM.gif]

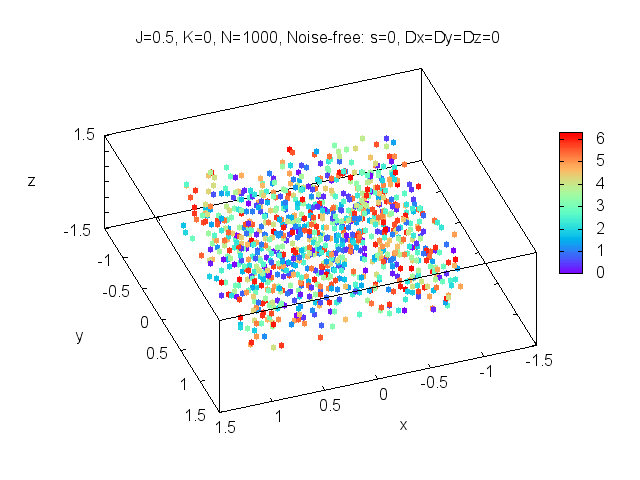

Supplement: Supplementary file 13 — Supplementary Movie 10 [file 41467_2017_1190_MOESM13_ESM.gif]

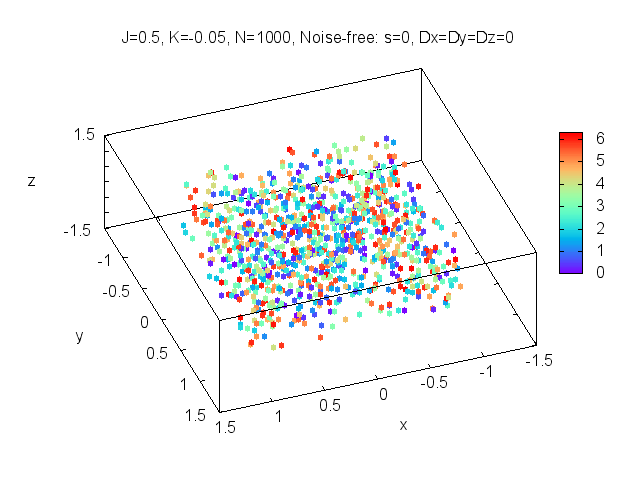

Supplement: Supplementary file 14 — Supplementary Movie 11 [file 41467_2017_1190_MOESM14_ESM.gif]

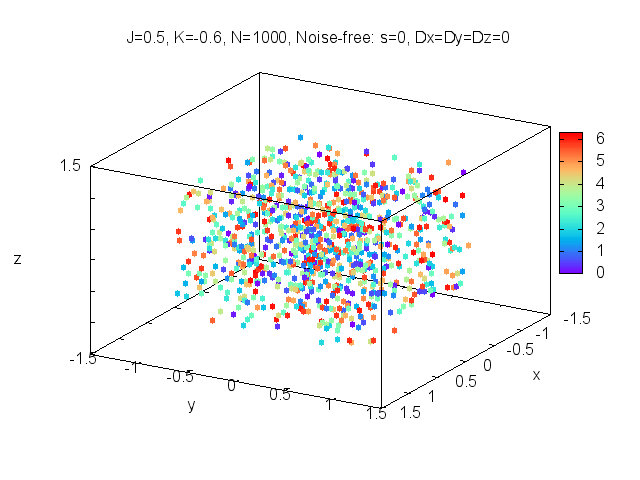

Supplement: Supplementary file 15 — Supplementary Movie 12 [file 41467_2017_1190_MOESM15_ESM.gif]
